# Decision-Theoretic Ecological Optimization of the Curzon–Ahlborn Heat Engine

**DOI:** 10.3390/e28091023

**Published:** 2026-09-14

**Authors:** Karen K. Huruntz, Lilit E. Ghukasyan, Ashok Vaseashta, Artak V. Gevorgyan

**Affiliations:** 1 Department of Biology, Yerevan State University, Yerevan 0025, Armenia; 2 Strategic Research, International Clean Water Institute, Manassas, VA 20108-0258, USA; 3 Institute of Mechanics, Yerevan 0019, Armenia

**Keywords:** Curzon–Ahlborn heat engine, finite-time thermodynamics, ecological optimization, Nash bargaining solution, minimax-regret optimization

## Abstract

Finite-time heat engines must balance useful power output against irreversibility. We develop a decision-theoretic framework for ecological optimization of the endoreversible Curzon–Ahlborn engine based on its normalized power–loss Pareto frontier. We first examine bargaining-based selection rules. For Newtonian heat transfer, a symmetric Nash compromise between retained power and reduced exergy destruction selects a unique interior operating point with efficiency $\eta_{N}=1-\tau^{3/4}$, where $\tau =T_{c}/T_{h}$ is the cold-to-hot reservoir temperature ratio. The same Newtonian state is recovered by the Arias-Hernández–Angulo-Brown prescription, which fixes the ecological weight from the maximum-power reference. Our contribution is therefore not a new efficiency law but a decision-theoretic interpretation of the established 3/4 law as a distinguished compromise between maximum-power operation and the reversible Carnot limit. Under the same normalization, the Kalai–Smorodinsky and egalitarian selectors also identify the same balance point. We then extend the bargaining analysis beyond Newton’s law of cooling and compare the Nash continuation with the Arias-Hernández–Angulo-Brown $E_{\epsilon }$ − $C\left(\epsilon \right)$ prescription. Although coinciding in the Newtonian limit, the two approaches separate at first order when the heat-transfer law is perturbed, thereby distinguishing their underlying selection principles. This comparison raises a further question: how should an operating state be chosen when the relative priority assigned to power and irreversibility reduction is itself uncertain? We address this question through minimax-regret optimization, which selects the operating point that minimizes the largest loss relative to the optimum that would have been chosen had the priority been known. Under complete normalized uncertainty in the ecological priority, the regret-robust selector again yields $\eta =1-\tau^{3/4}$, giving the same operating state a complementary interpretation in terms of robustness to priority uncertainty.

## 1. Introduction

The ideal Carnot engine establishes the upper bound on heat-engine efficiency, but this bound is reached only in the reversible limit where the cycle time diverges and the power output vanishes. Real heat engines therefore operate in a fundamentally finite-time regime, in which useful power must be extracted at the cost of finite thermodynamic forces and nonzero entropy generation. The central question of finite-time thermodynamics is therefore not simply how to maximize efficiency but how to choose an operating point that balances power production against irreversibility [1,2,3,4]. Recent work has substantially broadened this program through low-dissipation models [5], generalized endoreversible models [6,7], constrained optimal-control approaches [8], and multiobjective methods that construct tradeoffs among power, efficiency, ecological performance, and related objectives [9]; recent perspectives summarize these developments and wider directions in finite-time thermodynamics [10]. Against this background, the present work addresses a complementary decision-theoretic question: Once the thermodynamic tradeoff among competing objectives is known, what principle should select a particular operating state from it?

The Curzon–Ahlborn (CA) engine provides the canonical model of this compromise [11]. In its standard endoreversible form, the working substance is internally reversible, while irreversibility is confined to finite-rate heat exchange with the hot and cold reservoirs. For Newtonian heat transfer, maximizing the power output of the CA engine gives the well-known maximum-power efficiency [11,12,13,14] $\eta_{MP}=1-\sqrt{\tau }$, $\tau =T_{c}/T_{h}$, where $T_{h}$ and $T_{c}$ ($T_{h}>T_{c}$) denote the reservoir temperatures. This result is often interpreted as a natural finite-time alternative to Carnot efficiency. However, maximum power is only one possible operating principle. It privileges throughput and does not directly account for the useful work destroyed by entropy generation.

This observation motivated ecological and related optimization criteria. In these approaches, the engine is not judged by power alone but by a compromise between useful output and dissipation. A typical ecological objective has the form [15] $E=P-T_{c}{\dot{S}}_{gen}$ or, equivalently, $P-{\dot{X}}_{d}$, where ${\dot{X}}_{d}=T_{c}{\dot{S}}_{gen}$ is the Gouy–Stodola exergy-destruction rate [16,17]. Here P is the power output and ${\dot{S}}_{gen}$ is the entropy-generation rate. The appeal of such criteria is clear: entropy generation is not introduced as a merely formal penalty but as destroyed available work. Ecological optimization therefore shifts the selected operating point away from pure maximum power toward lower irreversibility.

At the same time, ecological criteria raise a second question. More generally, one may write a weighted ecological family [18] $J_{\lambda }=P-\lambda {\dot{X}}_{d}$, where λ≥0 measures the relative weight assigned to exergy destruction. If λ is externally fixed by economics, regulation, or design constraints, the problem is well posed. But if the goal is to formulate a thermodynamic selection principle, the weight itself requires justification. Otherwise, one has not eliminated arbitrariness; it has only shifted from the operating variable to the objective function.

The first purpose of this paper is to formulate ecological optimization as a Pareto-selection problem. In this formulation, the thermodynamic model determines the feasible tradeoff, while the choice of a particular operating point requires an additional decision criterion. The CA engine generates a curve of feasible operating states: moving toward the Carnot edge reduces exergy destruction but also reduces power, while moving toward maximum power increases output but increases irreversibility. Thus, the problem is structurally analogous to a bargaining problem: two competing objectives must be jointly improved, and a rule is needed to select one compromise point from the Pareto frontier.

Pareto and multiobjective formulations have previously been used in finite-time thermodynamics to characterize tradeoffs among power, efficiency, entropy generation, exergy destruction, and related objectives [19,20,21]. Bargaining and game-theoretic approaches have also been applied to thermodynamic and heat-engine problems [22,23]. The present use is more specific: the competing quantities are performance objectives of a single finite-time engine, and bargaining is employed as a rule for selecting an operating state from its thermodynamically generated Pareto frontier.

We adopt the Nash bargaining solution as this rule. In its simplest form, the Nash solution selects the feasible point that maximizes the product of gains above a reference point. For the heat engine, we take the two gains to be normalized power retention and normalized reduction in exergy destruction, $p=P/P_{MP}$ and $r=1-{\dot{X}}_{d}/{\dot{X}}_{d,MP}$, where $P_{MP}$ is the maximum power of the CA engine and ${\dot{X}}_{d,MP}$ is the exergy-destruction rate at maximum power. The proposed Nash ecological criterion is therefore N=pr. This product form penalizes one-sided operation: a point with high power but little irreversibility reduction or low irreversibility but negligible power is not optimal. The selected state must be good in both senses simultaneously.

For the Newtonian endoreversible CA model, this criterion gives a unique interior point with efficiency $\eta_{N}=1-\tau^{3/4}$, which lies between the CA maximum-power efficiency and the Carnot efficiency. This value is not new as a numerical efficiency in the finite-time thermodynamics literature; related ecological, modified ecological [18,24,25], and saving-function [26] approaches have also led to the same 3/4 law. The central question is therefore not the specific algebraic form of the already known 3/4 law but why this operating point constitutes a meaningful compromise between the maximum-power and reversible limits. The contribution here is the interpretation and closure: the same point arises from a Nash-type Pareto selector rather than from imposing an externally weighted scalar objective. Moreover, the Nash point can be mapped back to the weighted ecological family, yielding an endogenous effective weight $\lambda^{⋆}=\tau^{-1/2}$. Thus, the tradeoff parameter is not assumed; it is derived from the geometry of the compromise.

A second aim of the paper is to test whether this agreement persists when the heat-transfer law is perturbed away from the Newtonian form. We consider a generalized cooling law in which the heat flux is proportional to a power n of the temperature difference, with n=1+δ, where δ measures the departure from Newton’s law. In this setting, different selection principles that coincide at n=1 need not remain equivalent. In particular, we compare the Nash ecological selector with the Arias-Hernández–Angulo-Brown [18] construction based on the family $E_{\epsilon }=P-\epsilon T_{c}{\dot{S}}_{gen}$, where the parameter ϵ is selected by their $C\left(\epsilon \right)$ procedure. The two methods agree in the Newtonian limit n=1 but separate at first order in δ, providing a quantitative diagnostic for distinguishing ecological selection principles beyond the canonical CA limit.

Another decision problem arises when, within the weighted ecological family $J_{\lambda }=P-\lambda {\dot{X}}_{d}$, the relative priority assigned to power retention and reduction in exergy destruction is itself uncertain. If λ were known, the corresponding weighted objective would select a definite operating point. But when the relevant priority is not known in advance, optimization for one assumed value of λ may be fragile: the chosen state can perform poorly if the effective ecological preference differs from the assumed one.

We address this problem through minimax-regret optimization. Regret measures the loss incurred by selecting an operating point different from the one that would have been optimal had the priority been known. The minimax-regret rule then selects the state whose worst possible regret over an admissible priority interval is smallest. This does not replace the bargaining formulation. Nash bargaining selects a compromise when the two normalized objectives are placed on equal footing; minimax regret selects a regret-robust operating point when their relative importance is not known beforehand.

The full-uncertainty limit is especially revealing. When the admissible priority spans the entire normalized range, the regret of not operating at maximum power is the lost fraction of maximum power, whereas the regret of not eliminating exergy destruction is the remaining normalized exergy-destruction burden. Equalizing these two endpoint regrets again gives $\eta_{reg}=1-\tau^{3/4}$. The Newtonian 3/4 law therefore has two complementary decision-theoretic meanings: it is both a symmetric bargaining compromise and a regret-robust state under complete uncertainty about ecological priorities.

The paper is organized as follows. Section 2 introduces the endoreversible CA model and the power–loss Pareto branch. Section 3 formulates ecological optimization as a Pareto and bargaining problem. Section 4 derives the Nash ecological optimum for Newtonian heat transfer. Section 5 relates the Nash point to the weighted ecological family and to the Arias-Hernández–Angulo-Brown prescription. Section 6 extends the analysis to generalized heat transfer and compares the Nash and AHAB continuations. Section 7 examines alternative bargaining-based selectors. Section 8 formulates the minimax-regret criterion for uncertain ecological priorities. Section 9 treats the full-uncertainty limit and general uncertainty intervals. Section 10 provides conclusions and future directions.

## 2. Endoreversible CA Model and the Power–Loss Pareto Branch

We begin with the standard endoreversible Curzon–Ahlborn engine operating between two reservoirs at temperatures $T_{h}>T_{c}.$ The working substance is assumed to undergo an internally reversible cycle, while all irreversibility arises from finite-rate heat transfer across the hot and cold contacts. Let $T_{1}$ denote the effective temperature of the working substance during heat absorption from the hot reservoir, and let $T_{2}$ denote the corresponding temperature during heat rejection to the cold reservoir. Thus, $T_{h}>T_{1}>T_{2}>T_{c}$.

For Newtonian heat transfer, the heat currents are

(1) $$\begin{array}{c} {\dot{Q}}_{h}=K_{h}\left(T_{h}-T_{1}\right),\;\;\;\;\;\;{\dot{Q}}_{c}=K_{c}\left(T_{2}-T_{c}\right) \end{array},$$

where $K_{h}$ and $K_{c}$ are the hot- and cold-side thermal conductances. The endoreversibility condition means that the entropy gained by the working substance during heat uptake equals the entropy rejected during cooling:

(2) $$\frac{{\dot{Q}}_{h}}{T_{1}}=\frac{{\dot{Q}}_{c}}{T_{2}}.$$

This condition is what distinguishes the CA model from a fully irreversible engine. The engine is not globally reversible because heat exchange occurs through finite temperature differences, but the internal cycle itself is treated as reversible.

It is convenient to introduce the dimensionless temperature ratios:

(3) $$\tau =\frac{T_{c}}{T_{h}},\;\;\;\;x=\frac{T_{2}}{T_{1}}.$$

The engine efficiency is then

(4) $$\eta =1-\frac{T_{2}}{T_{1}}=1-x.$$

The Carnot value corresponds to the limiting case in which the working-fluid temperatures approach the reservoir temperatures, i.e., $T_{1}\to T_{h}$, $T_{2}\to T_{c}$; hence,

(5) $$x\to \tau ,\;\;\;\;\eta \to \eta_{C}=1-\tau .$$

Using Equations (1) and (2), one obtains the reduced CA power curve (detailed calculations are provided in Appendix A)

(6) $$P\left(x\right)=A\frac{\left(1-x\right)\left(x-\tau \right)}{x},$$

where

(7) $$A=T_{h}\;\frac{K_{h}K_{c}}{K_{h}+K_{c}}.$$

The prefactor A sets the power scale, while the dimensionless shape of the curve is controlled by τ. This separation is important: in the Newtonian endoreversible CA model, the conductances $K_{h}$ and $K_{c}$ affect the magnitude of power but not the location of the optimum in x.

Differentiating Equation (6) gives the maximum-power point $x_{MP}=\sqrt{\tau }$; therefore,

(8) $$\eta_{MP}=1-\sqrt{\tau }.$$

The corresponding maximum power is

(9) $$P_{MP}=A{\left(1-\sqrt{\tau }\right)}^{2}.$$

This is the familiar Curzon–Ahlborn result. It is the natural benchmark for throughput but not necessarily for ecological operation because it does not directly minimize irreversibility.

To describe irreversibility in a physically meaningful way, we use the Gouy–Stodola relation. Taking the cold-reservoir temperature $T_{c}$ as the environmental reference temperature, i.e., $T_{0}=T_{c}$, the exergy-destruction rate (equivalently, the Gouy–Stodola lost-work rate) is ${\dot{X}}_{d}=T_{c}{\dot{S}}_{gen}$, where ${\dot{S}}_{gen}$ is the total entropy-generation rate. For the present endoreversible engine, entropy is generated only by heat exchange with the reservoirs. The external entropy-generation rate is

(10) $${\dot{S}}_{gen}=-\frac{{\dot{Q}}_{h}}{T_{h}}+\frac{{\dot{Q}}_{c}}{T_{c}}.$$

Using the endoreversible relation and the definition of $x=T_{2}/T_{1}$, the exergy-destruction rate can be written in the same reduced variable as

(11) $${\dot{X}}_{d}(x)=A\frac{{\left(x-\tau \right)}^{2}}{x}.$$

At the maximum-power point,

(12) $${\dot{X}}_{d,MP}=A\sqrt{\tau }{\left(1-\sqrt{\tau }\right)}^{2}.$$

Equations (6) and (11) define the central power–loss structure of the paper. They show explicitly why ecological optimization is a Pareto problem. On the branch $x\in \left[\tau ,\sqrt{\tau }\right]$, moving from $x=\sqrt{\tau }$ toward x=τ increases efficiency and reduces exergy destruction but also lowers power. In the opposite direction, moving toward maximum power raises P but at the cost of a larger ${\dot{X}}_{d}$. Thus, no point on this branch dominates another: each represents a different compromise between useful output and destroyed available work.

For later use, we introduce the normalized variables

(13) $$p\left(x\right)=\frac{P\left(x\right)}{P_{MP}}$$

and

(14) $$r\left(x\right)=1-\frac{{\dot{X}}_{d}\left(x\right)}{{\dot{X}}_{d,MP}}.$$

Here, $p\left(x\right)$ measures the fraction of maximum power retained, while $r\left(x\right)$ measures the fraction of maximum-power exergy destruction avoided. If a distinct environmental reference temperature $T_{0}\neq T_{c}$ is adopted, the exergy-destruction rate is rescaled as ${\dot{X}}_{d}^{\left(0\right)}=T_{0}{\dot{S}}_{gen}=\left(T_{0}/T_{c}\right){\dot{X}}_{d}.$ Since the same factor multiplies both ${\dot{X}}_{d}$ and its maximum-power reference value, it cancels from the normalized gain. Thus, for a fixed environmental reference temperature, the normalized exergy-destruction coordinate and the normalized Pareto frontier are unchanged.

Definitions (13) and (14) are not arbitrary rescalings; they are endpoint normalizations of the physical Pareto branch so that each coordinate measures fractional progress across the full attainable range between the same two thermodynamic endpoint states. The maximum-power state is used here not as a uniquely privileged design point but as one endpoint of the Pareto branch; an application-specific design or operating reference would introduce additional external information and define a different bargaining specification. The endpoint normalization is adopted because its reference states are determined entirely by the CA model and require no additional application-specific baseline.

Explicitly,

(15) $$p\left(x\right)=\frac{\left(1-x\right)\left(x-\tau \right)}{x{\left(1-\sqrt{\tau }\right)}^{2}}$$

and

(16) $$r\left(x\right)=1-\frac{{\left(x-\tau \right)}^{2}}{\sqrt{\tau }\;x{\left(1-\sqrt{\tau }\right)}^{2}}.$$

For later use, we note that both normalized gains are strictly concave on the open ecological branch $x\in \left(\tau ,\sqrt{\tau }\right)$. Indeed,

(17) $$p^{\prime \prime }\left(x\right)=-\frac{2\tau }{x^{3}{\left(1-\sqrt{\tau }\right)}^{2}}<0$$

and

(18) $$r^{\prime \prime }\left(x\right)=-\frac{2\tau^{2}}{\sqrt{\tau }\;x^{3}{\left(1-\sqrt{\tau }\right)}^{2}}<0.$$

The two dimensionless quantities $p\left(x\right)$ and $r\left(x\right)$ provide a natural coordinate system on the CA Pareto branch. The point $x=\sqrt{\tau }$ corresponds to $\left(p,r\right)=\left(1,\;0\right)$, while the reversible edge x=τ corresponds to $\left(p,r\right)=\left(0,\;1\right)$.

**Figure 1:**
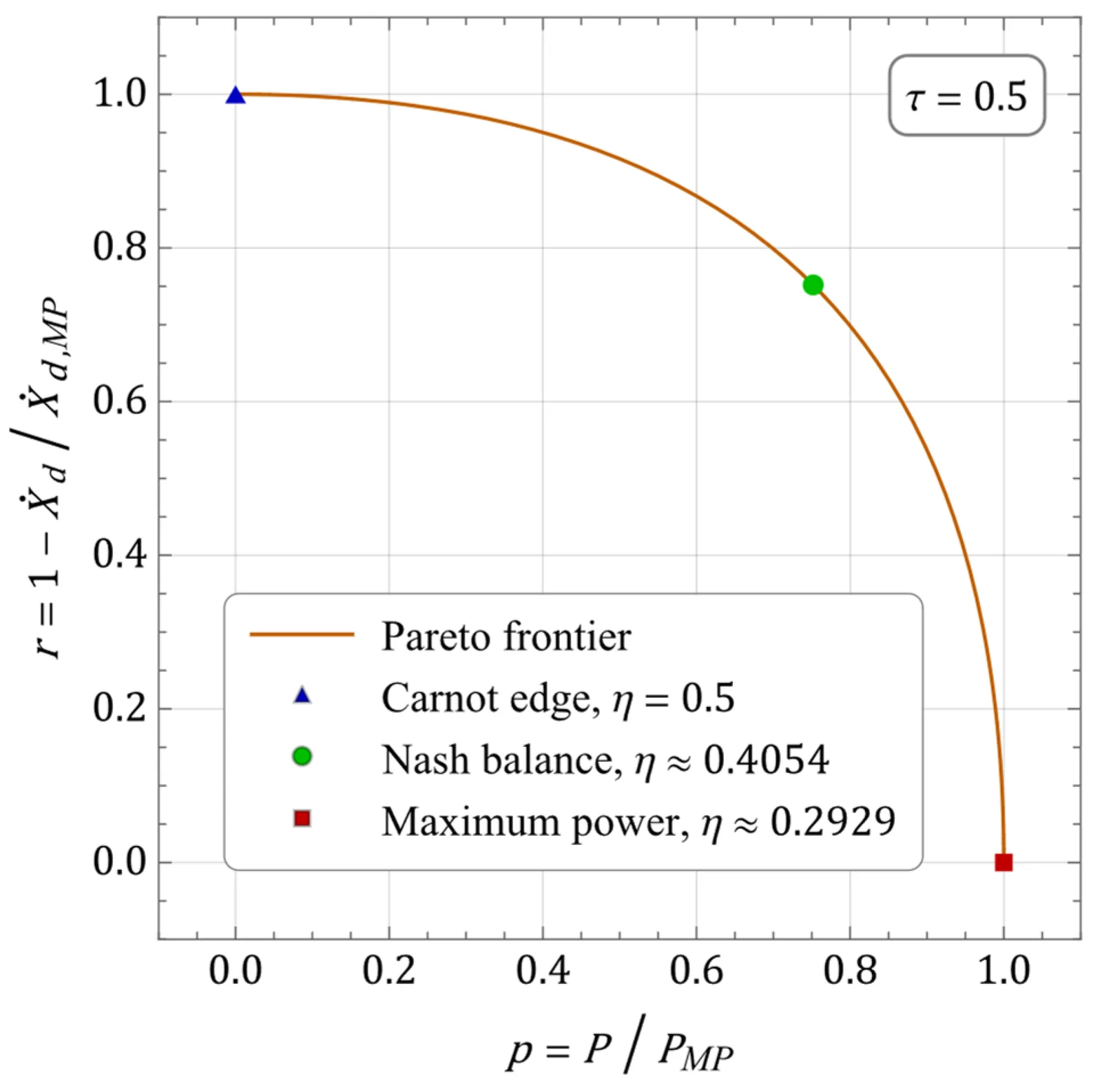
Normalized power–loss Pareto branch of the Newtonian Curzon–Ahlborn engine for τ=0.5. The vertical coordinate is avoided exergy destruction ($r=1-{\dot{X}}_{d}/{\dot{X}}_{d,MP}$), and the horizontal coordinate is retained power ($p=P/P_{MP}$). Maximum-power operation corresponds to $\left(p,r\right)=\left(1,0\right)$, while the reversible Carnot edge corresponds to $\left(p,r\right)=\left(0,1\right)$. The Nash, Kalai–Smorodinsky, and egalitarian selectors coincide at the balance point p=r, giving $\eta =1-\tau^{3/4}$.

The resulting normalized frontier is shown in Figure 1 for a representative value of τ=0.5. The figure makes explicit the Pareto structure: moving from maximum power toward the reversible edge increases the avoided exergy destruction r but decreases the retained power p.

The ecological optimization problem can now be stated cleanly: choose one point on this curve. Maximum power selects the left endpoint in $\left(p,r\right)$ space, Carnot operation selects the opposite endpoint, and a meaningful ecological criterion should select an interior compromise. The next section explains why bargaining theory, especially the Nash product, provides a natural way to make this selection.

## 3. Pareto Structure and Bargaining-Based Selection

The previous section showed that the Newtonian CA engine naturally generates a curve of operating states in the plane of normalized retained power p and normalized irreversibility reduction r. This representation makes the structure of the problem transparent. Maximum-power operation gives p=1 but r=0: all available power retention but no improvement in exergy destruction relative to the maximum-power reference. The Carnot edge gives r=1 but p=0: maximal irreversibility reduction but no power. Between these endpoints lies a continuum of finite-power, lower-loss compromises.

This is precisely the structure of a Pareto problem. A point on a feasible set is called Pareto optimal if no other feasible point improves one objective without worsening the other. On the CA ecological branch, $x\in \left[\tau ,\sqrt{\tau }\right],$ increasing r requires decreasing p, and increasing p requires decreasing r. Thus, the CA engine does not give one distinguished optimum by thermodynamics alone. It gives a Pareto frontier.

This distinction is important. Pareto optimality eliminates dominated operating states, but it does not tell us which non-dominated state should be chosen. The maximum-power point, the Carnot edge, and every intermediate point on the branch are Pareto efficient in the narrow sense. A further principle is required to select a unique ecological operating point.

The conventional way to select such a point is to introduce a weighted objective, for example [18],

(19) $$J_{\lambda }\left(x\right)=P\left(x\right)-\lambda {\dot{X}}_{d}\left(x\right).$$

This is mathematically useful and physically meaningful if λ is known. For instance, λ might encode fuel price, environmental regulation, carbon penalty, or design preference. But if the aim is to construct a purely thermodynamic ecological criterion, then λ becomes an additional unexplained parameter. In that case, the original optimization problem has not been fully closed; the arbitrariness has merely moved from the choice of operating point to the choice of scalarizing weight.

This is where bargaining theory becomes useful. A bargaining problem consists of a feasible set of possible outcomes and a disagreement point, representing the outcome obtained if no agreement is reached. The task is not to generate the Pareto frontier; that frontier is already given. The task is to choose one agreement point on it according to a rational selection rule.

In the present setting, the two bargaining participants represent distinct interests evaluated on the same feasible set of operating states. This is structurally the same as in an ordinary bargaining problem, where different agents assign utilities to a common set of possible agreements. Here, the common object of choice is the operating state x of a single heat engine, while the two utility coordinates are $u_{1}=p\left(x\right)$ and $u_{2}=r\left(x\right)$, representing retained power and avoided exergy destruction, respectively. These interests may be associated with distinct design stakeholders or, equivalently, treated as two competing engineering priorities. The Pareto boundary of the feasible bargaining region is the CA Pareto curve in the $\left(p,r\right)$ plane. Choosing an ecological operating point is therefore mathematically equivalent to choosing a bargaining agreement between the two competing performance interests.

Nash’s bargaining solution [27,28] is especially natural for this problem. It is useful here precisely because it is not just another arbitrary scalarization. It is an axiomatic prescription for selecting one point from a feasible Pareto set. In the standard two-person bargaining problem, one is given a feasible utility set F and a disagreement point $d=\left(d_{1},d_{2}\right)$. Nash asked which rule should select the agreement point $u^{⋆}\in F$ and showed that a small set of natural axioms leads uniquely to the product-of-gains solution

(20) $$u^{⋆}=\arg\underset{u\in F,\;u_{i}\geq d_{i}}{\max}\left(u_{1}-d_{1}\right)\left(u_{2}-d_{2}\right).$$

The relevant axioms are the following [27,29].

First, Pareto efficiency: the selected point should lie on the Pareto frontier, since otherwise both objectives could be improved simultaneously. In the heat-engine problem, this means the selected operating state should be on the power–loss tradeoff branch, not inside the dominated region.

Second, symmetry: if the two objectives are placed on equal footing, the selection rule should not favor one merely because of labeling. In the present problem, this equal treatment is a decision assumption rather than a consequence of normalization alone. The purpose of the bargaining construction is to identify a compromise between retained power and avoided exergy destruction without assigning either objective an a priori priority. Once both objectives have been expressed as dimensionless endpoint-normalized gains and in the absence of external engineering, economic, environmental, or regulatory information that would justify favoring one over the other, we therefore adopt the symmetric formulation as the neutral benchmark. An asymmetric Nash product would instead introduce such a preference before the compromise is selected. If unequal priorities are externally specified, they can be incorporated through the weighted Nash formulation considered in Section 7.3.

Third, invariance under positive affine rescaling of utilities: the selected point should not depend on arbitrary units or linear rescalings of the utility coordinates. This is especially important in thermodynamics, where raw quantities such as power, entropy generation, and efficiency have different dimensions and scales. By using normalized utilities p and r, the bargaining formulation avoids a spurious dependence on units. More specifically, positive affine transformations of either utility leave the Nash-selected operating state unchanged when the disagreement coordinates are transformed consistently; by contrast, changing the disagreement baseline or the relative weighting of the two objectives changes the bargaining problem and can shift the selected state.

Fourth, independence of irrelevant alternatives: once the selected point is feasible, removing unchosen alternatives that would not have been selected should not change the solution. In the present setting, this means the compromise is determined by the local structure of the attainable power–loss frontier rather than by arbitrary extensions of the feasible set that play no role in the selected agreement.

Together, these axioms make the Nash solution more than a convenient product objective. They turn it into a principled rule for closing a Pareto problem. The Nash product rewards balanced gains because if either $u_{1}-d_{1}$ or $u_{2}-d_{2}$ is small, the product remains small. Thus, a point with high power but almost no irreversibility reduction or a point with strong irreversibility reduction but negligible power is disfavored.

For the normalized CA engine, we take $u_{1}=p$ and $u_{2}=r$. The corresponding comprehensive feasible utility region is

(21) $$F=\left\{\left(u_{1},u_{2}\right)\in R_{+}^{2}:\exists x\in \left[\tau ,\sqrt{\tau }\right]\;\mathrm{such}\;\mathrm{that}\;0\leq u_{1}\leq p\left(x\right)\;\mathrm{and}\;0\leq u_{2}\leq r\left(x\right)\right\},$$

whose Pareto boundary is the physical CA operating curve $\left(p\left(x\right),r\left(x\right)\right)$. For 0<τ<1, F is compact and convex; the latter follows from the concavity of the Newtonian CA frontier in the $\left(p,r\right)$ plane. With the endpoint normalization introduced in Section 2, the natural disagreement point is the zero-gain reference $d=\left(0,\;0\right)$, corresponding to zero credited gain in both retained power and avoided exergy destruction. It belongs to F, and feasible points with both gains strictly above the disagreement level exist. Thus, the thermodynamic model supplies a feasible utility set of the standard form required for the Nash bargaining problem. The Nash axioms, by contrast, are properties imposed on the selection rule rather than additional physical assumptions on the heat engine. Any nonzero disagreement point requires an externally specified baseline and is therefore not determined by the CA thermodynamics alone; such generalized disagreement points are considered in Section 7.4. Since the Nash solution is Pareto efficient, maximizing the Nash product over F reduces to maximization over the physical CA Pareto boundary.

The Nash prescription therefore becomes

(22) $$N\left(x\right)=p\left(x\right)r\left(x\right).$$

This is the Nash ecological criterion used in the next section.

The physical meaning is direct. We do not maximize power alone, nor do we maximize reversibility alone. We maximize the joint gain in retained output and reduction in exergy destruction. In geometric terms, $N\left(x\right)$ is the area of the rectangle spanned by the origin and the point $\left(p\left(x\right),r\left(x\right)\right)$ in the normalized Pareto plane. The selected point is therefore the state with the largest balanced performance rectangle.

The use of Nash’s solution does not imply that it is the only possible rational selector. Rather, it provides one particularly transparent axiomatic closure. Comparing it with other selection rules, such as Kalai–Smorodinsky [30], egalitarian [31], or weighted Nash rules, helps separate features that are specific to Nash from structural consequences of the CA Pareto geometry.

The advantage of the bargaining formulation is therefore twofold. First, once the normalized gains, their zero-gain reference, and their symmetric treatment are specified, it introduces no additional weighting parameter in selecting a point from the CA Pareto frontier. Second, it supplies a clear interpretation of the selected ecological state: it is not merely the optimum of an arbitrary scalar function but the balanced agreement between two normalized thermodynamic gains.

## 4. Nash Ecological Optimum in the Newtonian CA Model

We now apply the bargaining prescription from the previous section to the explicit Newtonian CA Pareto branch. The normalized utilities were defined by Equations (15) and (16). The Nash ecological objective is

(23) $$N\left(x\right)=p\left(x\right)r\left(x\right),x\in \left[\tau ,\sqrt{\tau }\right].$$

The endpoints have a clear interpretation. At the maximum-power point ($x=\sqrt{\tau }$), one has p=1 and r=0, so N=0. At the Carnot edge (x=τ), one has p=0 and r=1, so, again, N=0. The Nash product is continuous on the closed physical interval $x\in \left[\tau ,\sqrt{\tau }\right]$ and therefore attains a global maximum. Since N=0 at both endpoints while N>0 in the interior, every global maximizer must be interior. This already captures the intended physical compromise: neither pure maximum power nor zero-loss reversible operation is selected.

To find the optimum, set $s=\sqrt{\tau }$, $\tau =s^{2}$. Then, $x\in \left[s^{2},s\right]$. Substitution of Equations (15) and (16) into Equation (23), followed by differentiation, gives

(24) $$\frac{dN}{dx}=\frac{\left(s^{3}-x^{2}\right)\left[-2x^{2}+\left(1+s+s^{2}+s^{3}\right)x-2s^{3}\right]}{s\;x^{3}{\left(1-s\right)}^{4}}.$$

The denominator is positive throughout the open interval $x\in \left(s^{2},s\right)$. The second factor in the numerator, i.e.,

(25) $$G\left(x\right)=-2x^{2}+\left(1+s+s^{2}+s^{3}\right)x-2s^{3},$$

is also positive on the same interval. To see this, note that $G^{\prime \prime }\left(x\right)=-4<0$, so G is concave. Its minimum on the closed interval must therefore occur at one of the endpoints. Direct evaluation gives

(26) $$G\left(s^{2}\right)=s^{2}{\left(1-s\right)}^{2}\left(1+s\right)>0$$

and

(27) $$G\left(s\right)=s{\left(1-s\right)}^{2}\left(1+s\right)>0.$$

Hence, the sign of dN/dx is controlled entirely by $s^{3}-x^{2}$. The derivative is positive for $x<s^{3/2}$ and negative for $x>s^{3/2}$. Therefore, the Nash product has a unique interior maximum at

(28) $$x_{N}=s^{3/2}=\tau^{3/4}.$$

The corresponding efficiency is

(29) $$\eta_{N}=1-\tau^{3/4}.$$

This is the central Newtonian result. The Nash ecological criterion selects an operating point strictly between the maximum-power and Carnot limits:

(30) $$1-\tau^{1/2}<1-\tau^{3/4}<1-\tau ,\;\;\;\;0<\tau <1.$$

Equivalently,

(31) $$\eta_{MP}<\eta_{N}<\eta_{C}.$$

**Figure 2:**
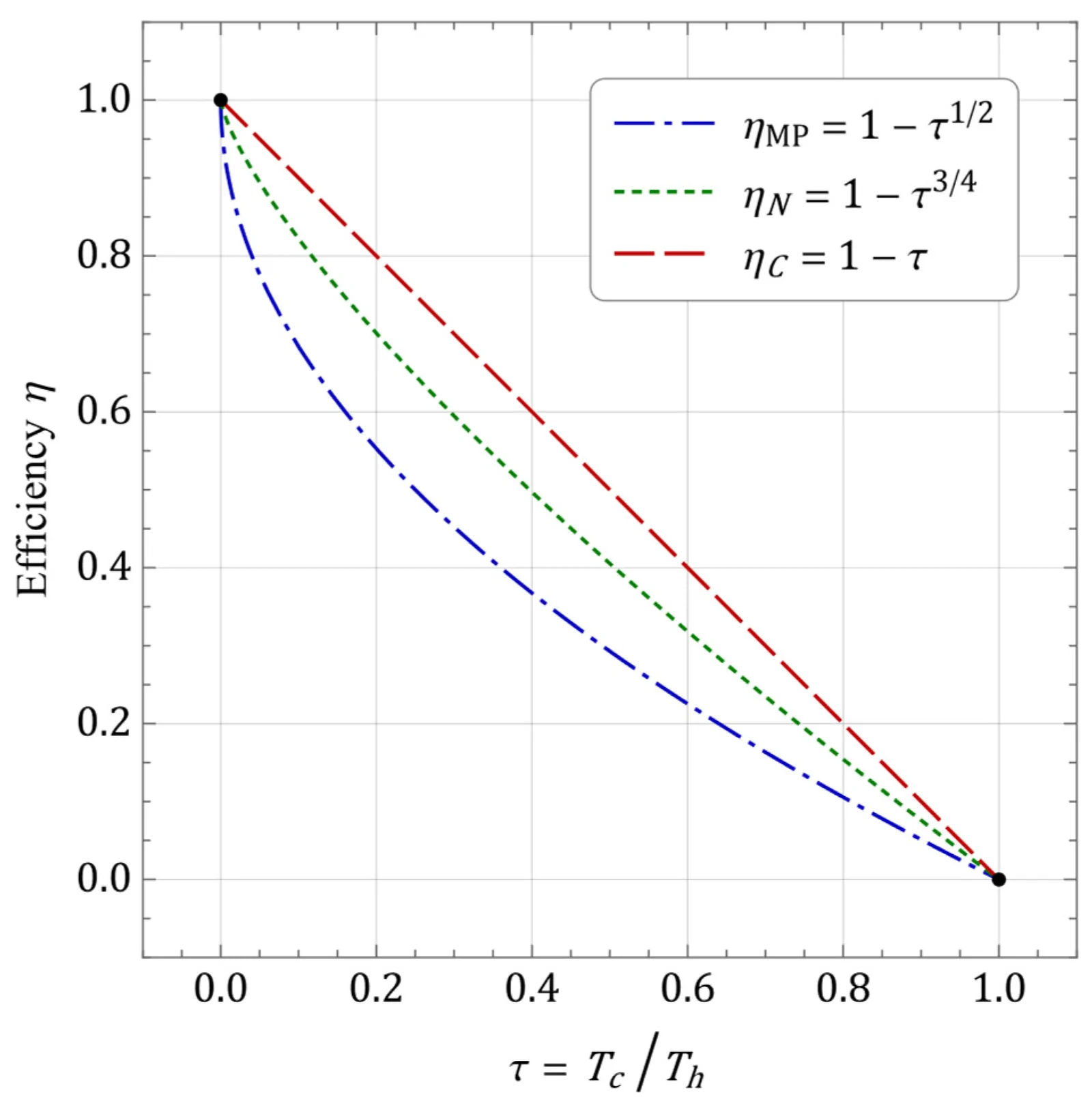
Efficiency benchmarks as functions of the reservoir temperature ratio $\tau =T_{c}/T_{h}$. The maximum-power Curzon–Ahlborn efficiency ($\eta_{MP}=1-\tau^{1/2}$), the Nash ecological efficiency ($\eta_{N}=1-\tau^{3/4}$), and the Carnot efficiency ($\eta_{C}=1-\tau$) are shown.

Figure 2 shows these three efficiencies as functions of τ. The Nash-selected curve lies between the maximum-power and Carnot curves throughout 0<τ<1.

Thus, the Nash ecological point is neither a small perturbation of maximum power nor an almost reversible operating state. It is a genuine interior compromise.

It is useful to examine the normalized performance at this point. Let $v=\tau^{1/4}$ so that $x_{N}=v^{3}$. Substitution into Equations (15) and (16) gives

(32) $$\frac{P_{N}}{P_{MP}}=\frac{1+v+v^{2}}{{\left(1+v\right)}^{2}}$$

and

(33) $$1-\frac{{\dot{X}}_{d,N}}{{\dot{X}}_{d,MP}}=\frac{1+v+v^{2}}{{\left(1+v\right)}^{2}}.$$

That is, at the Nash point,

(34) $$p\left(x_{N}\right)=r\left(x_{N}\right).$$

This equality is not imposed by the Nash criterion itself. More generally, maximizing the symmetric Nash product N=pr along a smooth Pareto frontier $r=r\left(p\right)$ gives

(35) $$\frac{dr}{dp}=-\frac{r}{p}.$$

Thus, Nash bargaining provides a specific marginal balance condition: at the selected state, the marginal tradeoff between the two normalized gains is balanced by the ratio of the gains already attained. For the normalized Newtonian CA frontier, the special geometry makes this Nash condition occur precisely at p=r. In thermodynamic terms, the retained fraction of maximum power then equals the fraction of maximum-power exergy destruction avoided. As discussed in Section 7, the Kalai–Smorodinsky condition with $d=\left(0,0\right)$ and an ideal point $U=\left(1,1\right)$ also reduces to p=r, while the egalitarian max–min rule selects the same intersection on the monotone Pareto frontier. Hence, the common 3/4 law reflects a distinguished symmetric point of the normalized Newtonian CA geometry rather than equivalence of the underlying selection principles. What Nash bargaining uniquely provides is the marginal product-of-gains balance by which this point is selected; this condition remains meaningful when the special geometry responsible for the coincidence is changed.

Near thermal equilibrium between the two reservoirs, i.e., in the weak-driving regime $\eta_{C}=1-\tau \ll 1$, the result has a simple interpretation. Expanding,

(36) $$\eta_{MP}=1-\sqrt{\tau }=\frac{\eta_{C}}{2}+O\left(\eta_{C}^{2}\right),$$

while

(37) $$\eta_{N}=1-\tau^{3/4}=\frac{3}{4}\eta_{C}+O\left(\eta_{C}^{2}\right).$$

Thus, in the linear-response regime, maximum power occurs at one-half Carnot efficiency, whereas the Nash ecological point occurs at three-quarters Carnot efficiency. This places the Nash point closer to reversible operation but still away from the zero-power Carnot edge.

For a representative value of τ=0.5, one obtains $\eta_{MP}\approx 0.2929$, $\eta_{N}\approx 0.4054$, and $\eta_{C}=0.5$. At the Nash point, the engine retains about three-quarters of its maximum-power output while reducing the maximum-power exergy-destruction burden to about one-quarter. This gives the 3/4 law a direct performance interpretation, not merely an algebraic one.

The result should also be positioned carefully relative to earlier ecological optimization literature. The efficiency $1-\tau^{3/4}$ has appeared before in modified ecological [18,24,25] and saving-function [26] approaches. The present contribution is not the first occurrence of this numerical expression. Rather, the point is that the same efficiency emerges from a bargaining-theoretic Pareto selector and therefore acquires a different interpretation: it is the symmetric Nash agreement between power retention and reduction in exergy destruction.

In the next section, we show that this Nash-selected point can also be represented within the weighted ecological family $P-\lambda {\dot{X}}_{d}$ but with the weight λ fixed endogenously by the Nash selection rather than chosen externally.

## 5. Relation to the AHAB Ecological Selection and Endogenous Weight

The previous section derived the Nash ecological point $x_{N}=\tau^{3/4}$, $\eta_{N}=1-\tau^{3/4}$. As noted above, this operating point is not numerically new. A closely related value appears in the Arias-Hernández–Angulo-Brown ecological program [18] and in the subsequent discussion with Yan [24,25], where the modified ecological family is written as $E_{\epsilon }=P-\epsilon T_{c}{\dot{S}}_{gen}$ and the parameter ϵ is selected by an auxiliary compromise criterion $C\left(\epsilon \right)$. In the exact Newtonian CA case, that construction selects the weight $\epsilon^{⋆}=\sqrt{T_{h}/T_{c}}=\tau^{-1/2}$ and, consequently, the same efficiency $\eta =1-\tau^{3/4}.$

The $E_{\epsilon }$ − $C\left(\epsilon \right)$ prescription is detailed in Appendix B.3, together with a lemma relating the $C\left(\epsilon \right)$-selected weight to the maximum-power state.

Thus, the purpose of the present section is to show how the same weight emerges from the Nash/Pareto geometry developed above. This makes explicit the relation between the two approaches: in the Newtonian CA limit, the AHAB C-selected ecological criterion and the symmetric Nash ecological selector choose the same Pareto point.

To see this connection, consider the weighted ecological family

(38) $$J_{\lambda }\left(x\right)=P\left(x\right)-\lambda {\dot{X}}_{d}\left(x\right),$$

where ${\dot{X}}_{d}=T_{c}{\dot{S}}_{gen}.$ Using Newtonian CA expressions (6) and (11), one obtains

(39) $$J_{\lambda }\left(x\right)=A\;\frac{\left(1-x\right)\left(x-\tau \right)-\lambda {\left(x-\tau \right)}^{2}}{x}.$$

The stationarity condition for an interior optimum is

(40) $$\left(1+\lambda \right)x^{2}=\tau \left(1+\lambda \tau \right),$$

so the weighted ecological family selects

(41) $$x\left(\lambda \right)=\sqrt{\frac{\tau \left(1+\lambda \tau \right)}{1+\lambda }}.$$

For λ=0, this gives the maximum-power point $x\left(0\right)=\sqrt{\tau }$. In the opposite limit, λ→∞, the selected point approaches the reversible edge, $x\left(\lambda \right)\to \tau .$ Thus, the family $P-\lambda {\dot{X}}_{d}$ parametrizes the same ecological Pareto branch between maximum power and Carnot operation.

Now, we impose the condition that this weighted optimum coincides with the Nash-selected point, i.e.,

(42) $$x\left(\lambda^{⋆}\right)=x_{N}=\tau^{3/4}.$$

Using Equation (41), this gives

(43) $$\tau^{3/4}=\sqrt{\frac{\tau \left(1+\lambda^{⋆}\tau \right)}{1+\lambda^{⋆}}}.$$

Solving for $\lambda^{⋆}$, one obtains

(44) $$\lambda^{⋆}=\tau^{-1/2}.$$

This is precisely the effective weight selected in the exact Newtonian AHAB construction. The point of the present derivation is therefore interpretive: the same coefficient is obtained as the weighted-sum representation of the Nash agreement between normalized power retention and reduction in exergy destruction.

In this sense, the Nash construction does not replace the AHAB ecological framework in the Newtonian CA case. Instead, it gives a decision-theoretic explanation for why the selected weight has this form. The AHAB route begins from the family $E_{\epsilon }=P-\epsilon T_{c}{\dot{S}}_{gen}$ and uses the compromise function $C\left(\epsilon \right)$ to determine ϵ. The Nash route begins with the normalized Pareto coordinates and selects the balanced product maximum. For Newtonian CA heat transfer, these two selection principles meet at the same point: $x^{⋆}=\tau^{3/4}$, $\eta =1-\tau^{3/4}$, $\lambda^{⋆}=\tau^{-1/2}$. The distinction becomes important only when one moves away from the exact Newtonian CA structure. If the heat-transfer law is generalized to $\dot{Q}\propto {\left(\Delta T\right)}^{n}$, n=1+δ, then these two approaches coincide at n=1, but they need not coincide at first order in δ. This is the reason we analyze the n=1+δ case in detail below. The comparison is not meant to show that one method is a trivial rephrasing of the other; rather, it shows that the Newtonian agreement is a special coincidence, while away from n=1, the two selection prescriptions generate different perturbative branches.

## 6. Generalized Heat Transfer and Comparison with AHAB Prescription

The Newtonian CA model is a special case in which several selection principles coincide. The Nash selector, the Kalai–Smorodinsky balance condition, the egalitarian balance condition, and the exact Newtonian Arias-Hernández–Angulo-Brown $E_{\epsilon }$ − $C\left(\epsilon \right)$ prescription all lead to $x^{⋆}=\tau^{3/4}$ and $\eta =1-\tau^{3/4}$.

The more interesting question is what happens when the physical model is perturbed. If two selection principles agree only because the Newtonian CA curve has a special algebraic structure, then their predictions should separate once that structure is deformed. To test this, we consider a generalized heat-transfer law close to Newton’s law [32]—namely, a symmetric generalized CA model with heat currents in the form of

(45) $${\dot{Q}}_{h}=a{\left(T_{h}-T_{1}\right)}^{n},\;\;\;\;\;\;{\dot{Q}}_{c}=a{\left(T_{2}-T_{c}\right)}^{n},$$

where n=1+δ, $\left|\delta \right|\ll 1$. Here, δ measures the departure from Newtonian heat transfer. The Dulong–Petit neighborhood corresponds to positive δ, with the common idealized value of n=5/4, i.e., δ=1/4.

The symmetric case is sufficient for isolating the effect of the selection rule itself. Conductance asymmetry can be included, but it adds parameters without changing the conceptual point.

For this power-law heat-transfer model, the reduced power and exergy-destruction factor can be written, up to an irrelevant positive prefactor, as

(46) $${\hat{P}}_{n}\left(x\right)=\frac{\left(1-x\right){\left(x-\tau \right)}^{n}}{x{\left[1+x^{\left(n-1\right)/n}\right]}^{n}}$$

and

(47) $${\hat{\Sigma }}_{n}\left(x\right)=\frac{{\left(x-\tau \right)}^{n+1}}{x{\left[1+x^{\left(n-1\right)/n}\right]}^{n}}.$$

Here, ${\hat{\Sigma }}_{n}$ denotes the reduced quantity proportional to the exergy-destruction rate, ${\dot{X}}_{d}=T_{c}{\dot{S}}_{gen}$. These expressions reduce to the Newtonian CA forms at n=1.

A useful identity remains valid for this whole class:

(48) $$\frac{{\hat{P}}_{n}\left(x\right)}{{\hat{\Sigma }}_{n}\left(x\right)}=\frac{1-x}{x-\tau }.$$

This identity is important because it is the basis of the AHAB ecological-weight construction [18]. It says that the ratio of useful power to exergy-destruction penalty depends only on the operating point x, not explicitly on the exponent n.

The technical details of what follows are collected in Appendix B.

### 6.1. Maximum-Power Point for n=1+δ

The maximum-power point is defined by

(49) $$\frac{\partial {\hat{P}}_{n}\left(x\right)}{\partial x}=0.$$

Let $t=\tau^{1/4}$ and $\sqrt{\tau }=t^{2}$. At Newtonian order, $x_{MP}^{\left(0\right)}=\sqrt{\tau }=t^{2}.$ Expanding about n=1 gives

(50) $$x_{MP}=t^{2}+\delta \;\frac{1-t^{4}}{4}+O\left(\delta^{2}\right).$$

Therefore,

(51) $$\eta_{MP}=1-t^{2}-\delta \;\frac{1-t^{4}}{4}+O\left(\delta^{2}\right).$$

For δ>0, the maximum-power efficiency shifts downward. This is already a first indication that the n=1 CA formulas should not be treated as universal once the heat-transfer law is changed.

### 6.2. AHAB Continuation: Ecological Family with $C\left(\epsilon \right)$-Selected Weight

The Arias-Hernández–Angulo-Brown ecological construction is based on the weighted family

(52) $$E_{\epsilon }\left(x;n\right)={\hat{P}}_{n}\left(x\right)-\epsilon {\hat{\Sigma }}_{n}\left(x\right),$$

where ϵ is not meant to remain arbitrary. In their $C\left(\epsilon \right)$-selection procedure, the optimal weight is determined from the maximum-power reference (see Lemma A1 in Appendix B.3). In the present notation, this can be written as

(53) $$\epsilon^{⋆}\left(n\right)=\frac{P_{MP}}{{\left(T_{c}{\dot{S}}_{gen}\right)}_{MP}}=\frac{{\hat{P}}_{n}\left(x_{MP}\right)}{{\hat{\Sigma }}_{n}\left(x_{MP}\right)}.$$

Using the identity (48), this becomes

(54) $$\epsilon^{⋆}\left(n\right)=\frac{1-x_{MP}}{x_{MP}-\tau }.$$

Thus, the AHAB algorithm can be understood in two steps: first, find the maximum-power point $x_{MP}\left(n\right)$; second, use it to fix $\epsilon^{⋆}\left(n\right)$, then maximize $E_{\epsilon^{⋆}}\left(x;n\right)$.

Substituting Equation (50) into Equation (54) gives

(55) $$\epsilon^{⋆}\left(n\right)=\tau^{-1/2}-\delta \;\frac{{\left(1+\sqrt{\tau }\right)}^{2}}{4\tau }+O\left(\delta^{2}\right).$$

At δ=0, this reduces to $\epsilon^{⋆}\left(1\right)=\tau^{-1/2}$, which is the same effective weight identified in the Newtonian Nash representation. This is the precise sense in which the two approaches coincide at the Newtonian benchmark n=1.

Now, let the AHAB-selected operating point be

(56) $$x_{AHAB}=t^{3}+\delta A_{1}\left(\tau \right)+O\left(\delta^{2}\right).$$

Maximizing $E_{\epsilon^{⋆}}\left(x;n\right)$ gives

(57) $$A_{1}\left(\tau \right)=\frac{t}{8}\left(1+2t-2t^{3}-t^{4}\right).$$

Therefore,

(58) $$\eta_{AHAB}=1-t^{3}-\delta A_{1}\left(\tau \right)+O\left(\delta^{2}\right).$$

Equation (58) is the AHAB prediction near Newtonian heat transfer. For δ>0, since $A_{1}\left(\tau \right)>0$ for 0<τ<1, the selected efficiency decreases relative to the Newtonian 3/4 law.

### 6.3. Nash Continuation for n=1+δ

The strict Nash continuation is different. Instead of first converting the problem into a weighted ecological objective, we recompute the normalized Pareto utilities for each n:

(59) $$p\left(x;n\right)=\frac{{\hat{P}}_{n}\left(x\right)}{{\hat{P}}_{n}\left(x_{MP}\right)},\;\;\;\;\;\;r\left(x;n\right)=1-\frac{{\hat{\Sigma }}_{n}\left(x\right)}{{\hat{\Sigma }}_{n}\left(x_{MP}\right)}.$$

The Nash ecological objective is then $N\left(x;n\right)=p\left(x;n\right)r\left(x;n\right)$. The Nash-selected point is defined by $\partial N\left(x;n\right)/\partial x=0$. At n=1, this gives $x_{N}^{\left(0\right)}=t^{3}$. For n=1+δ, we write

(60) $$x_{N}=t^{3}+\delta N_{1}\left(\tau \right)+O\left(\delta^{2}\right).$$

The coefficient $N_{1}\left(\tau \right)$ is more complicated than the AHAB coefficient $A_{1}\left(\tau \right)$ because the Nash objective contains the full n-dependent normalizations ${\hat{P}}_{n}\left(x_{MP}\right)$ and ${\hat{\Sigma }}_{n}\left(x_{MP}\right)$. The explicit closed form is given in Appendix B. The resulting efficiency is

(61) $$\eta_{N}=1-t^{3}-\delta N_{1}\left(\tau \right)+O\left(\delta^{2}\right).$$

The key point is not the algebraic complexity itself but the fact that $N_{1}\left(\tau \right)\neq A_{1}\left(\tau \right)$. The Nash and AHAB prescriptions agree at n=1, but their perturbative continuations are distinct.

### 6.4. Difference Between the Two Selections

Subtracting Equation (58) from Equation (61), we obtain

(62) $$\eta_{N}-\eta_{AHAB}=\delta \left[A_{1}\left(\tau \right)-N_{1}\left(\tau \right)\right]+O\left(\delta^{2}\right).$$

For the symmetric model considered here, the coefficient $A_{1}\left(\tau \right)-N_{1}\left(\tau \right)$ changes sign once, at $\tau_{0}\approx 0.02458$. For the physically common range $\tau >\tau_{0}$, the Nash continuation predicts a slightly higher efficiency than the AHAB continuation for δ>0, while for very large temperature contrasts $\tau <\tau_{0}$, the ordering is reversed.

Thus, the Nash selector predicts a slightly different efficiency than the AHAB C-selected ecological criterion when the transfer law is deformed in the Dulong–Petit direction. The difference is small but systematic. At n=1, the Newtonian CA curve has a special algebraic structure that makes several selection rules coincide. Once n≠1, the feasible Pareto curve changes shape, and the rules are no longer equivalent.

**Figure 3:**
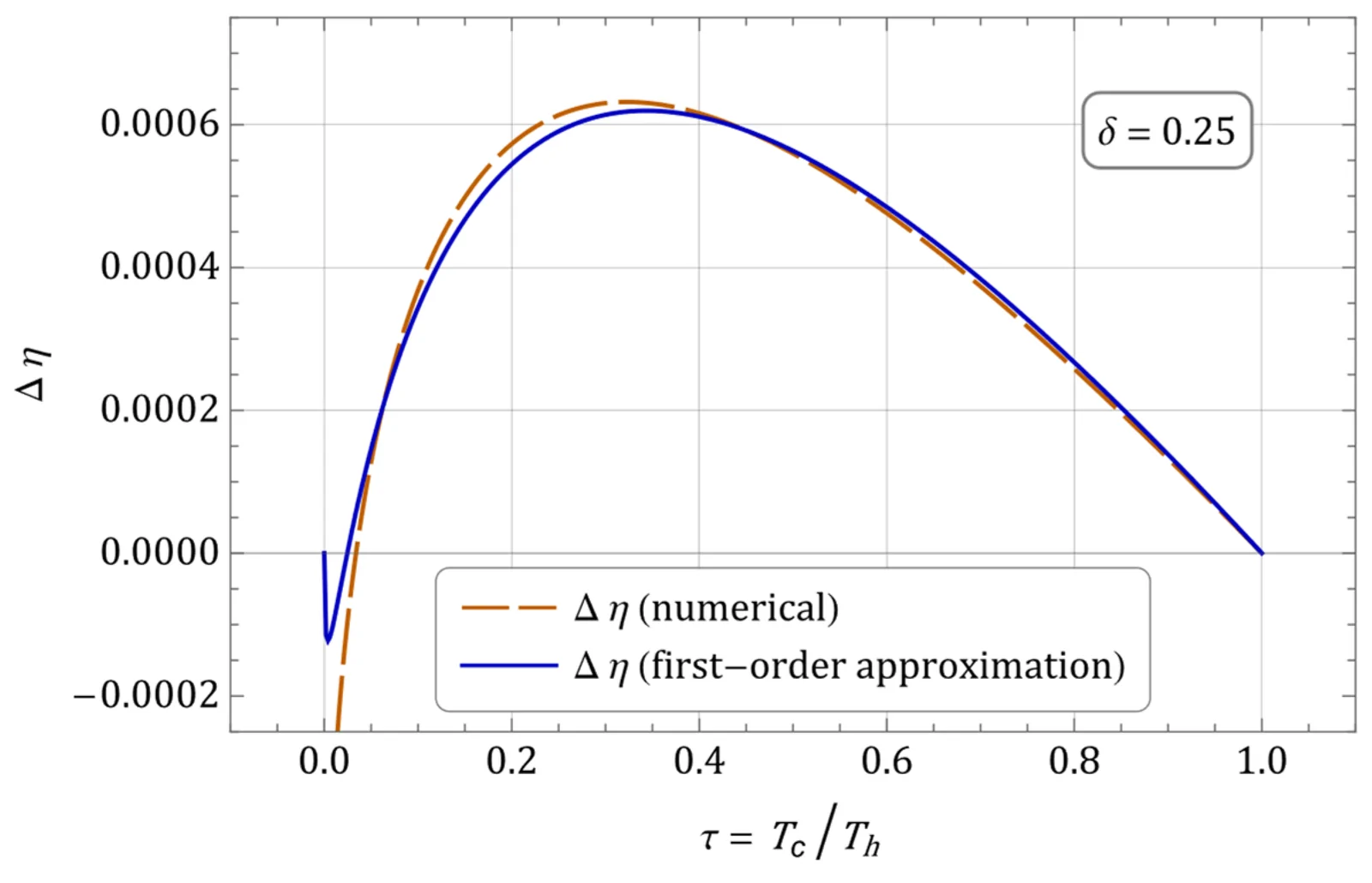
Difference $\Delta \eta =\eta_{N}-\eta_{AHAB}$ between the Nash and AHAB selected efficiencies for the generalized heat-transfer exponent n=1.25 (δ=0.25). The dashed curve is obtained by direct numerical optimization of the full n-dependent model, while the solid curve shows the first-order approximation of Equation (62). The two results agree closely over most of the reservoir-temperature range; the largest visible deviations occur for small τ values, where the exact zero crossing shifts from the perturbative value of $\tau_{0}≃0.02458$ to $\tau_{0}≃0.03387$.

Figure 3 compares the direct numerical difference $\Delta \eta \left(\tau \right)=\eta_{N}-\eta_{AHAB}$ obtained by optimizing the full n-dependent model with the first-order approximation for the Dulong–Petit value of n=1.25 (δ=0.25). The two curves agree closely over most of the reservoir-temperature range, confirming both the small magnitude and the systematic character of the Nash–AHAB separation predicted perturbatively. Over 0.1≤τ≤0.9, the exact difference reaches a maximum of approximately $6.32\times 10^{-4}$ near τ≃0.32, corresponding to about 0.063 percentage points in efficiency. For comparison, at the larger deviation of δ=0.5 (n=1.50), the maximum exact separation over the same τ range increases to approximately $\Delta \eta_{max}≃1.26\times 10^{-3}$ near τ≃0.30, corresponding to about 0.126 percentage points in efficiency. The main deviation occurs at a very small τ: the exact sign reversal is shifted to $\tau_{0}≃0.03387$, compared with the first-order value of $\tau_{0}≃0.02458$. Thus, the first-order treatment remains quantitatively accurate for n=1.25 over the practically relevant range while becoming less reliable in the limit of very large reservoir-temperature contrast.

A numerical scan over 0.1≤τ≤0.9 further shows that the first-order Nash and AHAB selected efficiencies remain within approximately 1% of the exact numerical values up to n≃1.35. Over this range, direct global optimization of the full n-dependent objectives over the physical operating interval yields a unique admissible optimum for each prescription, with no competing local maxima. By n=1.50, the maximum error has increased to about 2.1%, indicating the onset of appreciable higher-order corrections.

The comparison clarifies the role of the Newtonian result. The equality $\eta_{N}=\eta_{AHAB}=1-\tau^{3/4}$ at n=1 should not be read as showing that the Nash and AHAB methods are identical. Rather, it shows that the Newtonian CA model is a common benchmark where the two prescriptions meet. Both are reasonable ecological selection rules. They coincide for Newtonian CA heat transfer, but they define different continuations for n=1+δ. This is why the numerical and perturbative comparison is useful: it separates a shared Newtonian benchmark from the underlying selection principles that generate it.

## 7. Alternative Pareto Selectors and Selector Robustness of the 3/4 Law

The Nash product is the central selector used in this work, but it is not the only rational way to choose a point on a Pareto frontier. Since the CA ecological branch is a simple one-dimensional frontier connecting maximum power to the reversible edge, it is useful to ask whether the result $\eta =1-\tau^{3/4}$ is specific to Nash bargaining or instead exhibits selector robustness in the sense of being preserved under other symmetric Pareto-selection rules.

This question is useful because ecological optimization is not only a matter of writing down a scalar objective. Once the power–loss tradeoff is represented as a Pareto curve, different axiomatic or decision-theoretic selectors can be applied to the same thermodynamic frontier. Nash selection is one such rule; Kalai–Smorodinsky [30], egalitarian [31], weighted Nash, and baseline-dependent bargaining prescriptions are others.

For the normalized Newtonian CA frontier used here, the symmetric alternatives give a particularly simple result. With utilities defined by retained power $p=P/P_{MP}$ and avoided exergy destruction $r=1-{\dot{X}}_{d}/{\dot{X}}_{d,MP}$, and with the adopted zero-gain reference $d=\left(0,\;0\right)$, the Kalai–Smorodinsky and egalitarian selectors reduce to the balance condition p=r. The symmetric Nash product also selects this same balance point. Therefore, all three prescriptions identify the same Newtonian operating state, i.e., $\eta =1-\tau^{3/4}.$

This agreement supports the interpretation of the 3/4 law as a structural balance point of the normalized CA Pareto frontier rather than as an artifact of one particular product objective.

The agreement is not universal, however. Once asymmetry or nonzero baselines are introduced, the selectors generally separate. A weighted Nash criterion biases the compromise toward either power retention or irreversibility reduction. A nonzero disagreement point can encode externally specified engineering requirements such as a minimum acceptable power fraction or a minimum ecological improvement. Unlike the zero-gain reference $d=\left(0,0\right)$ induced by the endpoint normalization, such nonzero baselines require additional application-specific information.

These extensions are not needed to derive the central Newtonian result, but they clarify the role of the Nash framework. Nash is not being presented as the only possible Pareto selector. Rather, it is a natural symmetric selector whose prediction is shared by several other symmetric rules in the Newtonian CA model, while controlled departures arise when priorities or externally specified baselines are introduced asymmetrically.

### 7.1. Kalai–Smorodinsky Selector

The Kalai–Smorodinsky (KS) bargaining solution [30] selects the Pareto point that preserves proportional gains relative to an ideal point U. For a two-objective feasible set with disagreement point d, the KS condition is

(63) $$\frac{u_{1}-d_{1}}{U_{1}-d_{1}}=\frac{u_{2}-d_{2}}{U_{2}-d_{2}}.$$

In the present normalized setting, the natural ideal point is $U=\left(1,\;1\right)$, while the adopted zero-gain reference is $d=\left(0,0\right)$.

Then, the KS condition (63) reduces to $p\left(x\right)=r\left(x\right)$. Using Equations (15) and (16), this condition becomes

(64) $$\frac{\left(1-x\right)\left(x-\tau \right)}{x{\left(1-\sqrt{\tau }\right)}^{2}}=1-\frac{{\left(x-\tau \right)}^{2}}{\sqrt{\tau }\;x{\left(1-\sqrt{\tau }\right)}^{2}}.$$

Let $s=\sqrt{\tau }$. After multiplying by $x{\left(1-s\right)}^{2}$, Equation (64) simplifies to

(65) $$\left(1-x\right)\left(x-s^{2}\right)=x{\left(1-s\right)}^{2}-\frac{{\left(x-s^{2}\right)}^{2}}{s}.$$

This reduces to $x^{2}=s^{3}$. Therefore $x_{KS}=s^{3/2}=\tau^{3/4}$, and

(66) $$\eta_{KS}=1-\tau^{3/4}.$$

Thus, with the normalization used in this paper, the KS solution selects the same operating point as the symmetric Nash product.

### 7.2. Egalitarian or Max–Min Selector

The egalitarian selector maximizes the smaller of the two gains:

(67) $$x_{eg}=\arg\underset{x\in \left[\tau ,\sqrt{\tau }\right]}{\max}\min\left\{p\left(x\right),r\left(x\right)\right\}.$$

Along the ecological CA branch, $p\left(x\right)$ decreases monotonically from unity to zero, whereas $r\left(x\right)$ increases monotonically from zero to unity. The maximum of the smaller utility therefore occurs at their crossing point, i.e., $p\left(x\right)=r\left(x\right)$. Using the result already obtained for the KS condition, $x_{eg}=\tau^{3/4}$. Therefore,

(68) $$\eta_{eg}=1-\tau^{3/4}.$$

Hence, for $d=\left(0,\;0\right)$ and $U=\left(1,\;1\right)$, Nash, Kalai–Smorodinsky, and egalitarian selection all identify the same balanced Pareto point.

### 7.3. Weighted Nash Selector

The weighted or asymmetric Nash selector is

(69) $$N_{\gamma }\left(x\right)={p\left(x\right)}^{\gamma }{r\left(x\right)}^{1-\gamma },\;\;0<\gamma <1.$$

The parameter γ gives a controlled way to bias the selection toward one of the two objectives.

For an interior optimum, $d\ln N_{\gamma }\left(x\right)/dx=0$, or equivalently,

(70) $$\gamma \frac{p^{\prime }\left(x\right)}{p\left(x\right)}+\left(1-\gamma \right)\frac{r^{\prime }\left(x\right)}{r\left(x\right)}=0.$$

This equation expresses equality of weighted relative marginal changes along the Pareto branch.

Using Equations (15) and (16), Equation (70) becomes

(71) $$-\frac{1}{x}+\gamma \left[\frac{1}{x-\tau }-\frac{1}{1-x}\right]+\left(1-\gamma \right)\frac{\sqrt{\tau }{\left(1-\sqrt{\tau }\right)}^{2}-2\left(x-\tau \right)}{\sqrt{\tau }{\left(1-\sqrt{\tau }\right)}^{2}x-{\left(x-\tau \right)}^{2}}=0.$$

The scalar Equation (71) determines the weighted Nash point $x_{\gamma }$. Although it may contain several formal roots, the admissible solution is unique on the physical interval $x\in \left(\tau ,\sqrt{\tau }\right)$. Indeed, for 0<γ<1, p>0 and r>0 in the interior, and both normalized gains are strictly concave. Hence, $\ln N_{\gamma }=\gamma \ln p+\left(1-\gamma \right)\ln r$ is strictly concave on this interval. Since $N_{\gamma }=0$ at both endpoints and $N_{\gamma }>0$ in the interior, it follows that, for every 0<τ<1 and 0<γ<1, the weighted Nash criterion possesses a unique global interior maximizer. Equivalently, $x_{\gamma }$ is the unique admissible root of Equation (71) that maximizes $N_{\gamma }$. In general, it is simplest to determine this root numerically. The limiting behavior is clear: $x_{\gamma }\to \sqrt{\tau }$ as γ→1, whereas $x_{\gamma }\to \tau$ as γ→0.

Thus, weighted Nash selection continuously interpolates between the maximum-power point and the reversible edge. The symmetric point γ=1/2 gives

(72) $$x_{1/2}=\tau^{3/4},\;\;\;\;\;\;\eta_{1/2}=1-\tau^{3/4}.$$

**Figure 4:**
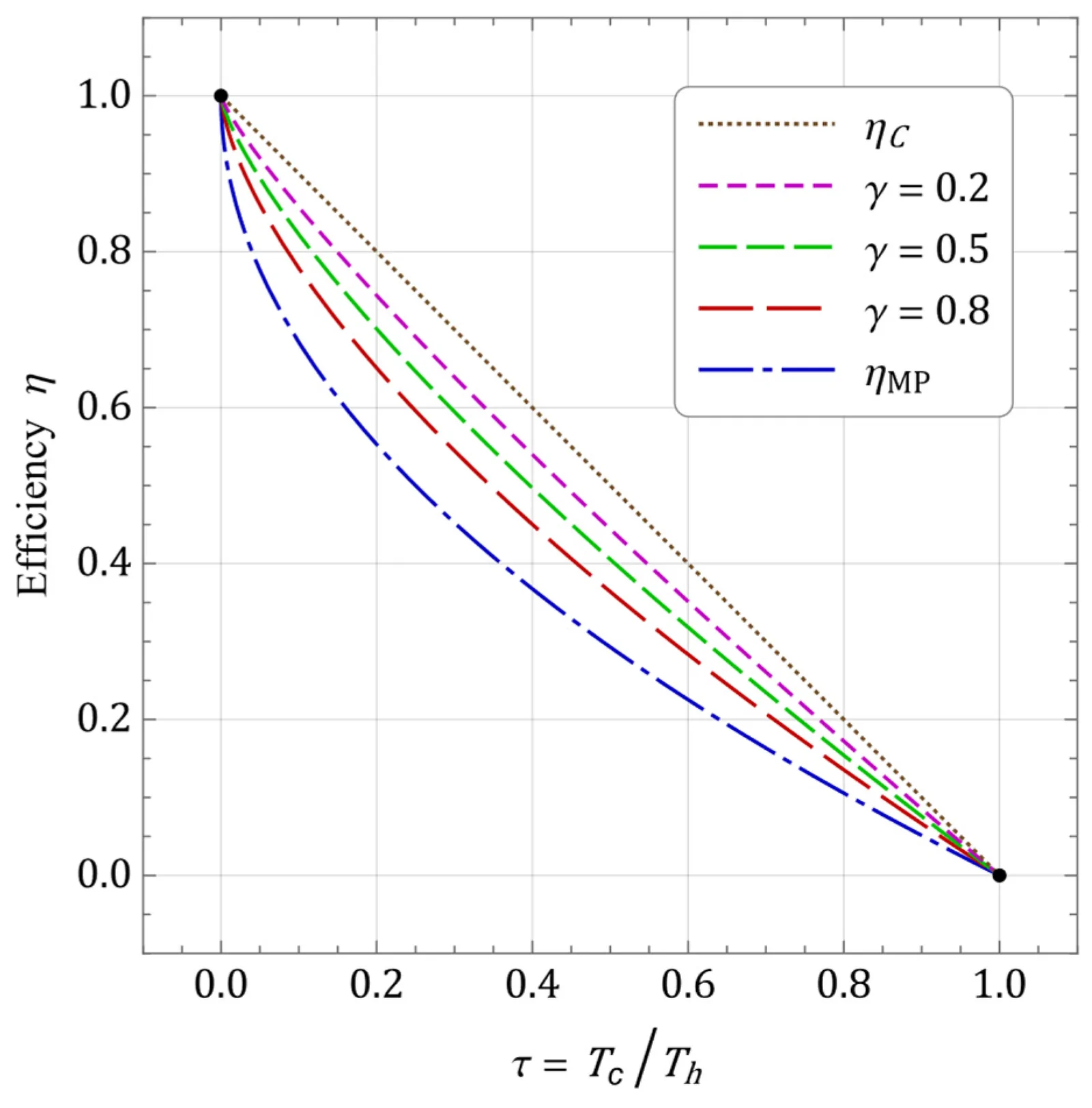
Weighted Nash selected efficiencies as functions of the reservoir temperature ratio $\tau =T_{c}/T_{h}$. The curves show the efficiency selected by the weighted Nash criterion $N_{\gamma }=p^{\gamma }r^{1-\gamma }$ for γ=0.2, 0.5, 0.8, together with the maximum-power efficiency ($\eta_{MP}=1-\tau^{1/2}$) and the Carnot limit ($\eta_{C}=1-\tau$). Increasing γ gives greater priority to power retention and shifts the selected efficiency toward $\eta_{MP}$, while decreasing γ gives greater priority to irreversibility reduction and shifts the selected efficiency toward $\eta_{C}$.

**Figure 5:**
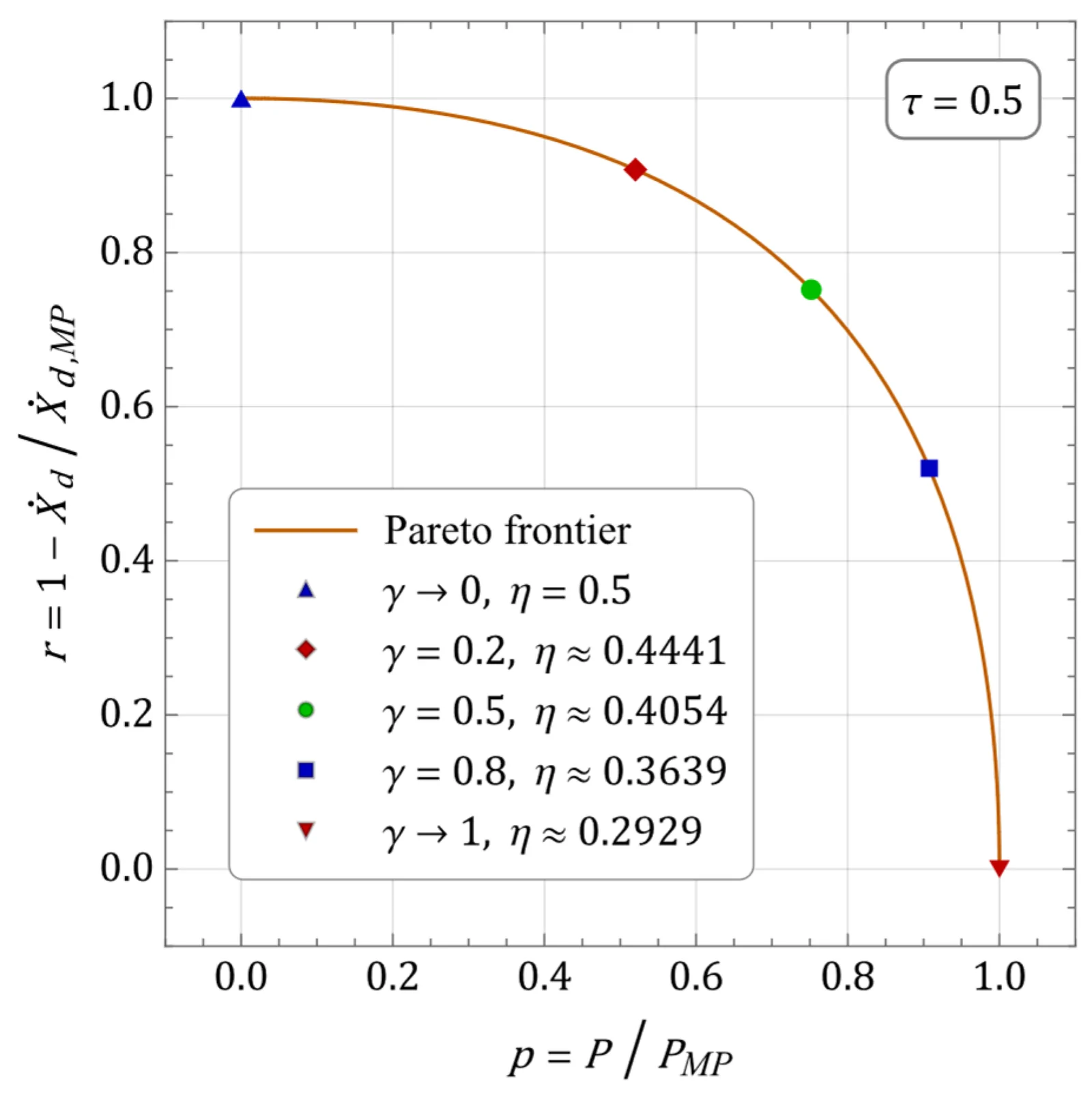
Weighted Nash operating points on the normalized Curzon–Ahlborn Pareto frontier for $\tau =T_{c}/T_{h}=0.5$. The horizontal axis shows retained power ($p=P/P_{MP}$), while the vertical axis shows avoided exergy destruction ($r=1-{\dot{X}}_{d}/{\dot{X}}_{d,MP}$). The orange curve is the Pareto frontier connecting maximum-power operation $\left(p,r\right)=\left(1,0\right)$ to the reversible Carnot edge $\left(p,r\right)=\left(0,1\right)$. Symbols mark the operating points selected by the weighted Nash criterion $N_{\gamma }=p^{\gamma }r^{1-\gamma }$. As γ→1, the selector approaches maximum power, while as γ→0, it approaches the Carnot edge. The symmetric case γ=0.5 gives the Nash balance point, i.e., $\eta =1-\tau^{3/4}\approx 0.4054$.

Figure 4 shows the selected efficiencies as functions of τ. Figure 5 shows the corresponding displacement of the operating point along the normalized Pareto frontier.

### 7.4. General Disagreement Points

The agreement of the Nash, Kalai–Smorodinsky, and egalitarian selectors above is obtained for the zero-gain reference $d=\left(0,\;0\right)$ associated with the endpoint normalization. More generally, one may consider a disagreement point $d=\left(d_{p},d_{r}\right)$, where $d_{p}$ and $d_{r}$ specify baseline gains in retained power and avoided exergy destruction, respectively. Unlike the zero-gain reference, nonzero disagreement coordinates are not determined by the CA thermodynamics alone and require externally specified baseline information, such as a minimum acceptable power fraction or a minimum ecological improvement.

For an externally prescribed disagreement point $d=\left(d_{p},d_{r}\right)$, the Nash product becomes

(73) $$N_{d}\left(x\right)=\left[p\left(x\right)-d_{p}\right]\left[r\left(x\right)-d_{r}\right],$$

defined on the feasible portion of the Pareto branch where $p\left(x\right)>d_{p}$ and $r\left(x\right)>d_{r}$. An interior optimum satisfies $d\ln N_{d}\left(x\right)/dx=0$. Using Equations (15) and (16), this condition can be written as

(74) $$\sqrt{\tau }\;\left(\tau -x^{2}\right)\left[r\left(x\right)-d_{r}\right]=\left(x^{2}-\tau^{2}\right)\left[p\left(x\right)-d_{p}\right].$$

Whenever the feasible portion of the Pareto branch is nonempty, the Nash product is positive in its interior and vanishes at its boundary. Moreover, $\ln N_{d}\left(x\right)=\ln\left[p\left(x\right)-d_{p}\right]+\ln\left[r\left(x\right)-d_{r}\right]$ is strictly concave on this interval. Hence, the stationarity condition has a unique admissible solution, which is the unique global maximum of $N_{d}$.

The corresponding Kalai–Smorodinsky condition, with an ideal point $U=\left(1,\;1\right)$, is

(75) $$\frac{p\left(x\right)-d_{p}}{1-d_{p}}=\frac{r\left(x\right)-d_{r}}{1-d_{r}},$$

whereas the egalitarian condition is

(76) $$p\left(x\right)-d_{p}=r\left(x\right)-d_{r}.$$

These conditions are generally different. Thus, for a general externally specified disagreement point, distinct bargaining rules need not select the same operating point. This observation is useful conceptually. The coincidence of the symmetric selectors at $d=\left(0,\;0\right)$ reflects the special normalized geometry of the Newtonian CA frontier. As shown below, this coincidence is preserved for symmetric disagreement baselines ($d_{p}=d_{r}$), whereas asymmetric baselines generally separate the selectors. Nonzero disagreement points allow bargaining theory to encode engineering constraints, such as a minimum acceptable power fraction, a minimum ecological improvement, or a status-quo operating mode.

An important special case is a symmetric disagreement baseline, i.e., $d_{p}=d_{r}=d_{0}$. The normalized Newtonian CA frontier has an exact exchange symmetry, which follows directly from Equations (15) and (16). With $s=\sqrt{\tau }$,

(77) $$p\left(x\right)=\frac{1+s^{2}-x-s^{2}/x}{{\left(1-s\right)}^{2}},\;\;\;r\left(x\right)=\frac{1+s^{2}-x/s-s^{3}/x}{{\left(1-s\right)}^{2}}.$$

Under the transformation $x\mapsto s^{3}/x$, which maps the ecological branch $x\in \left[s^{2},s\right]$ onto itself, these coordinates satisfy

(78) $$p\left(\frac{s^{3}}{x}\right)=r\left(x\right),\;\;\;r\left(\frac{s^{3}}{x}\right)=p\left(x\right).$$

Hence, the normalized Newtonian CA Pareto frontier is invariant under the interchange of p↔r. The transformation is involutive, and its unique fixed point on the ecological branch satisfies $x=s^{3}/x$, giving $x=s^{3/2}=\tau^{3/4}$, where p=r. A symmetric disagreement baseline preserves this exchange symmetry. Consequently, provided that the baseline is feasible, the Nash, Kalai–Smorodinsky, and egalitarian selectors continue to identify the balance point of $p\left(x\right)=r\left(x\right)$, $x=\tau^{3/4}$; hence, $\eta =1-\tau^{3/4}$.

Thus, the 3/4 law does not depend specifically on the numerical choice of $d=\left(0,\;0\right)$; it is preserved when the disagreement baseline treats the two normalized gains symmetrically.

By contrast, an asymmetric disagreement point, i.e., $d_{p}\neq d_{r}$, breaks this symmetry and shifts the selected operating state. A larger power baseline $d_{p}$ shifts the Nash compromise toward maximum-power operation and lowers the selected efficiency, whereas a larger baseline in avoided exergy destruction $d_{r}$ shifts it toward the reversible edge and raises the selected efficiency. The three bargaining selectors also no longer coincide in general. For example, at τ=0.5, the zero-gain reference $\left(d_{p},d_{r}\right)=\left(0,\;0\right)$, gives $\eta_{N}≃0.4054$. The choices of $\left(0.2,\;0\right)$ and $\left(0,\;0.2\right)$ give $\eta_{N}≃0.3964$ and 0.4143, respectively, whereas the symmetric choice of $\left(0.2,\;0.2\right)$ leaves the selected efficiency unchanged at $\eta_{N}≃0.4054$.

The disagreement-point analysis therefore distinguishes the zero-gain reference $d=\left(0,\;0\right)$ induced by the adopted endpoint normalization from application-specific nonzero baselines. Symmetric baseline changes preserve the Newtonian 3/4 law, whereas asymmetric baselines generate controlled departures from it.

## 8. Minimax-Regret Ecological Optimization Under Uncertain Priorities

The problem of bargaining optimization of heat engines arises from the need to replace the externally assigned relative priority between power retention and reduction in exergy destruction within the weighted ecological family $J_{\lambda }=P-\lambda {\dot{X}}_{d}$ by a thermodynamic selection principle. Another problem arises when this relative priority is imposed externally but is defined only vaguely rather than by a unique value of λ.

In the first case, the weighted ecological family defines a continuum of possible compromises but provides no intrinsic basis for selecting one of them. Bargaining formulations resolve this indeterminacy by choosing a point directly from the normalized power–loss Pareto frontier. The two gains may be treated symmetrically, assigned a specified asymmetry, or evaluated relative to an externally specified nonzero disagreement baseline.

In the second case, the relative priority has an external meaning, for example, through engineering, economic, or regulatory considerations—but is known only within a range of plausible values. The issue is then not the absence of a preference structure but uncertainty within an externally meaningful one. Minimax-regret optimization addresses this latter problem by selecting the operating state whose performance is least vulnerable to an incorrect specification of that priority.

### 8.1. Normalized Ecological Objective and Fixed-Priority Optima

The normalized objective used below is inspired by the ecological criterion $P-T_{c}{\dot{S}}_{gen}$ proposed by Angulo-Brown [15] and later generalized by Arias-Hernández and Angulo-Brown [18] to $P-\epsilon T_{c}{\dot{S}}_{gen}$. Their central idea is to judge a finite-time heat engine not by power alone but by a compromise between useful power output and the availability destroyed by entropy generation. Our modification is not to change this physical idea but to rewrite it in normalized variables suitable for regret analysis.

The reason is that regret depends on the numerical scale of the objective. Before introducing uncertainty in the tradeoff weight, we therefore normalize both power and exergy destruction by their respective values at maximum-power operation. This leads to the bounded weighted objective

(79) $$J_{\theta }\left(x\right)=\left(1-\theta \right)p\left(x\right)-\theta q\left(x\right),\;\;\;0\leq \theta \leq 1,$$

where

(80) $$q\left(x\right)=\frac{{\dot{X}}_{d}\left(x\right)}{{\dot{X}}_{d,MP}}=1-r\left(x\right)$$

is the normalized exergy-destruction burden. The parameter θ distributes a unit total priority between the two objectives. When θ=0, the objective is pure power retention, i.e., $J_{0}=p$. When θ=1, the objective is pure exergy-destruction minimization, i.e., $J_{1}=-q$. Intermediate values represent normalized tradeoffs between retaining power and reducing exergy destruction.

This normalized form is directly related to the conventional weighted ecological family $P-\epsilon {\dot{X}}_{d}$. For 0≤θ<1, maximizing Equation (79) is equivalent to maximizing $P-\epsilon_{\theta }{\dot{X}}_{d}$, with

(81) $$\epsilon_{\theta }=\frac{\theta }{1-\theta }\frac{P_{MP}}{{\dot{X}}_{d,MP}}=\frac{\theta }{\left(1-\theta \right)\sqrt{\tau }}.$$

Thus, the normalized objective is not a different physical tradeoff; it is the same power–loss scalarization expressed in dimensionless, bounded-weight form. This is important for regret analysis because regret depends on the scale of the objective. The variable $\theta \in \left[0,\;1\right]$ gives a normalized and bounded way to represent uncertainty in the relative priority of power and reduction in exergy destruction.

For completeness, we also record the operating point selected by $J_{\theta }$ when θ is known. For 0≤θ<1, maximizing $J_{\theta }$ on the Newtonian CA frontier, Equations (15) and (16), gives, with $s=\sqrt{\tau }$,

(82) $$x^{⋆}\left(\theta \right)={\left[s^{3}\frac{1-\theta +\theta s}{s\left(1-\theta \right)+\theta }\right]}^{1/2}.$$

This fixed-weight optimization is the normalized counterpart of the ecological [15] and modified-ecological [18,24,25] optimization problems.

At equal normalized weighting, i.e., θ=1−θ=1/2, Equation (82) gives $x^{⋆}\left(1/2\right)=\tau^{3/4}$; hence, $\eta =1-\tau^{3/4}$. Thus, the AHAB modified-ecological 3/4 law appears as the equal-weight point of the normalized power–loss scalarization. This observation separates the underlying physical balance from the particular algorithm used to select a weight. The essential idea is already present in Angulo-Brown’s original ecological criterion [15]: optimize a compromise between power output and exergy destruction rather than power alone. In its dimensional form, however, this compromise is not an equal-weight comparison of normalized power retention and normalized reduction in exergy destruction because the result depends on the relative scales of P and ${\dot{X}}_{d}$. Rewriting the same idea in normalized variables gives the equal-weight objective p−q, equivalent to $J_{\theta }$ at θ=1/2. For the Newtonian CA frontier, this immediately selects $x^{⋆}=\tau^{3/4}$. The later Arias-Hernández–Angulo-Brown construction reaches the same law through a more elaborate two-stage procedure based on a weighted ecological family and a compromise function $C\left(\epsilon \right)$ [18,24,25]. Another route to the same 3/4 law was provided by Velasco et al. [26], who introduced saving functions associated with fuel consumption and thermal pollution. The normalized formulation used here displays the same balance more directly, although it is introduced primarily because it is the natural form for a minimax-regret analysis.

In the minimax-regret formulation below, the fixed-θ optima play the role of benchmark states: regret measures the loss incurred when one chooses a single operating point without knowing which value of θ will be relevant.

### 8.2. Minimax-Regret Criterion for Uncertain Priorities

The minimax-regret criterion is a decision rule for problems in which the relevant state of uncertainty is not known in advance [33,34,35,36]. Instead of asking which decision gives the largest payoff for one assumed state, it asks how much is lost relative to the decision that would have been optimal after the state is known. The selected decision is then the one whose worst such loss, or regret, is as small as possible.

In the present problem, the uncertain “state” is not an external thermodynamic state of the reservoirs. The reservoir temperatures are assumed to be fixed. Rather, the uncertainty concerns the effective ecological weight θ in the normalized objective (79). If θ were known, one would simply choose the corresponding fixed-θ optimum $x^{⋆}\left(\theta \right)$. If only an interval of admissible values is known, however, then optimizing for one chosen value of θ may be fragile. The minimax-regret criterion asks instead for the operating point that is least vulnerable to the wrong choice of ecological priority.

We therefore assume that $\theta \in \left[\theta_{-},\theta_{+}\right]$, with $0\leq \theta_{-}<\theta_{+}\leq 1$. For each fixed θ, the best operating point is $x^{⋆}\left(\theta \right)$, and the corresponding optimal value is

(83) $$J_{\theta }^{⋆}=\underset{x}{\max}J_{\theta }\left(x\right)=J_{\theta }\left(x^{⋆}\left(\theta \right)\right).$$

If one chooses an arbitrary operating point x, while the relevant preference priority is θ, the regret is

(84) $$R\left(x;\theta \right)=J_{\theta }^{⋆}-J_{\theta }\left(x\right).$$

Thus, $R\left(x;\theta \right)\geq 0$, with equality only when $x=x^{⋆}\left(\theta \right)$. Regret measures the loss relative to the operating point that would have been chosen if θ had been known in advance.

The minimax-regret operating point is defined by

(85) $$x_{reg}=\arg\underset{x}{\min}\underset{\theta \in \left[\theta_{-},\theta_{+}\right]}{\max}R\left(x;\theta \right).$$

This is the regret-robust ecological state. It minimizes the worst loss caused by uncertainty in the relative priority of power retention and reduction in exergy destruction.

The endpoint structure follows from the affine dependence of $J_{\theta }$ on θ. Since $J_{\theta }\left(x\right)$ is affine in θ for every fixed x, the optimized value $J_{\theta }^{⋆}=\underset{x}{\max}J_{\theta }\left(x\right)$ is the upper envelope of a family of affine functions of θ and is consequently convex in θ. Since $R\left(x;\theta \right)=J_{\theta }^{⋆}-J_{\theta }\left(x\right)$, the regret is also convex in θ for a fixed x. Therefore, the worst regret over a closed interval occurs at one of the endpoints:

(86) $$\underset{\theta \in \left[\theta_{-},\theta_{+}\right]}{\max}R\left(x;\theta \right)=\max\left\{R\left(x;\theta_{-}\right),R\left(x;\theta_{+}\right)\right\}.$$

Thus, the continuum of possible weights reduces to a two-endpoint regret problem.

The endpoint reduction is the first simplification. The second step concerns how the upper envelope $\max\left\{R\left(x;\theta_{-}\right),R\left(x;\theta_{+}\right)\right\}$ is minimized. In general, the minimizer need not occur at a crossing of the two regret curves. It may also occur at a boundary of the admissible interval or at an interior point where one endpoint regret alone is active and has a local minimum. In the regular balanced case used below, however, the relevant minimizer is an interior point at which both endpoint regrets are active. The upper envelope is then minimized at their crossing so that

(87) $$R\left(x_{reg};\theta_{-}\right)=R\left(x_{reg};\theta_{+}\right).$$

The Newtonian CA frontier considered below realizes this regular balanced structure, so the following derivation uses the equal-regret condition.

Substituting the definition of regret into Equation (87) gives

(88) $$J_{\theta_{-}}^{⋆}-J_{\theta_{-}}\left(x_{reg}\right)=J_{\theta_{+}}^{⋆}-J_{\theta_{+}}\left(x_{reg}\right).$$

Using Equation (79), this becomes

(89) $$J_{\theta_{-}}^{⋆}-J_{\theta_{+}}^{⋆}=\left(\theta_{+}-\theta_{-}\right)\left[p\left(x_{reg}\right)+q\left(x_{reg}\right)\right].$$

Hence, in the regular balanced case, the minimax-regret point is characterized by

(90) $$p\left(x_{reg}\right)+q\left(x_{reg}\right)=K\left(\theta_{-},\theta_{+}\right),$$

where

(91) $$K\left(\theta_{-},\theta_{+}\right)=\frac{J_{\theta_{-}}^{⋆}-J_{\theta_{+}}^{⋆}}{\theta_{+}-\theta_{-}}.$$

This is the central structural result. The regret-robust operating point is not obtained by choosing a representative value of θ. Instead, in the regular balanced case, it is the point whose value of p+q balances the regrets associated with the two endpoints of the uncertainty interval.

The parameter K should not be regarded as an arbitrary free parameter. It is determined by the uncertainty interval and by the fixed-θ optimal branch. Since p and q are normalized quantities satisfying 0≤p≤1 and 0≤q≤1 on the chosen power–loss tradeoff branch, one has 0≤p+q≤2, and therefore, $0\leq K\left(\theta_{-},\theta_{+}\right)\leq 2$. This bound follows from the normalization, not from any special algebraic property of a specific engine model. The particular engine model enters only when the condition $p\left(x_{reg}\right)+q\left(x_{reg}\right)=K$ is solved for the operating point.

The quantity K also has a useful interpretation. By the envelope theorem [37], the derivative of the optimized value of $J_{\theta }^{⋆}=J_{\theta }\left(x^{⋆}\left(\theta \right)\right)$ with respect to θ is obtained by differentiating $J_{\theta }$ explicitly with respect to θ while holding $x=x^{⋆}\left(\theta \right)$ fixed. Since the stationarity condition eliminates the implicit contribution from $dx^{⋆}/d\theta$, one obtains

(92) $$\frac{dJ_{\theta }^{⋆}}{d\theta }=-\left[p\left(x^{⋆}\left(\theta \right)\right)+q\left(x^{⋆}\left(\theta \right)\right)\right].$$

Therefore,

(93) $$K\left(\theta_{-},\theta_{+}\right)=\frac{1}{\theta_{+}-\theta_{-}}\int_{\theta_{-}}^{\theta_{+}}\left[p\left(x^{⋆}\left(\theta \right)\right)+q\left(x^{⋆}\left(\theta \right)\right)\right]d\theta .$$

Thus, K is the average value of p+q along the fixed-θ optimal branch over the uncertainty interval. In the regular balanced case, the minimax-regret point is the operating state whose own p+q equals this average.

The next section first considers complete normalized uncertainty, i.e., $\theta \in \left[0,\;1\right]$, for which the endpoint regrets acquire an especially transparent physical meaning. It then returns to general uncertainty intervals.

## 9. Full-Uncertainty Limit and General Uncertainty Intervals

### 9.1. Full-Uncertainty Limit and Recovery of the 3/4 Law

The most transparent case is the full uncertainty interval, i.e., $\theta \in \left[0,\;1\right].$ This interval means that, before the operating point is chosen, the relative priority between power retention and exergy-destruction minimization is completely unspecified. The two endpoints have simple meanings. At θ=0, the objective is $J_{0}\left(x\right)=p\left(x\right)$, so the optimal value is $J_{0}^{⋆}=1$, attained at the maximum-power point. The regret is therefore

(94) $$R\left(x;0\right)=1-p\left(x\right).$$

This is the lost fraction of maximum power.

At the other endpoint, θ=1, the objective is $J_{1}\left(x\right)=-q\left(x\right)$. Since $q\left(x\right)\geq 0$ and $q\left(\tau \right)=0$, the optimal value is $J_{1}^{⋆}=0$, attained at the reversible edge. The corresponding regret is

(95) $$R\left(x;1\right)=q\left(x\right).$$

This is the remaining normalized exergy-destruction burden.

**Figure 6:**
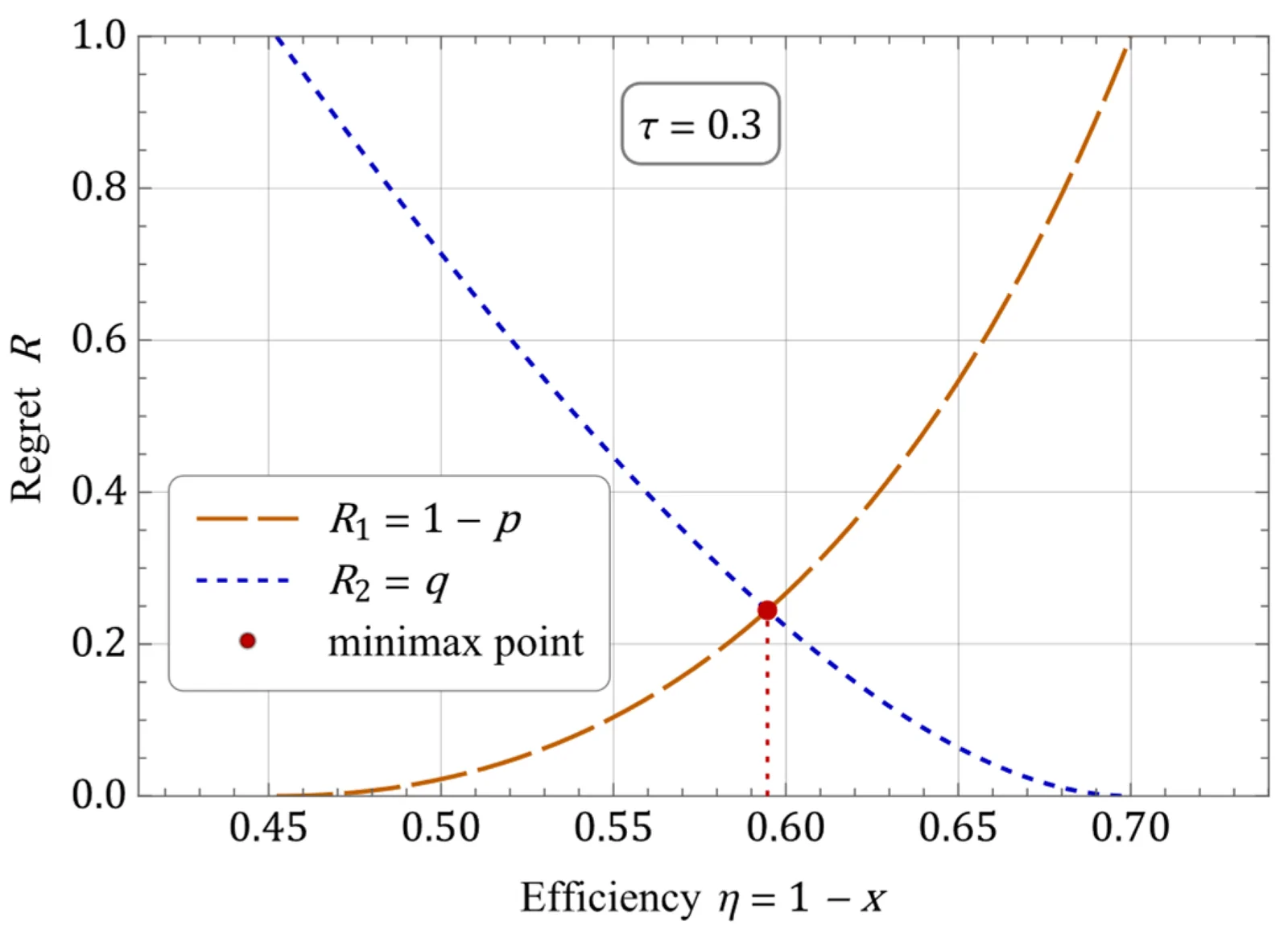
Full-uncertainty minimax-regret selection. For complete normalized uncertainty, i.e., θ∈[0,1], the endpoint regrets are $R\left(x;0\right)=1-p\left(x\right)$ and $R\left(x;1\right)=q\left(x\right)$. The minimax-regret point is obtained at their intersection, where the upper envelope $R_{max}\left(x\right)=\max\left\{R\left(x;0\right),R\left(x;1\right)\right\}$ is minimized. For the Newtonian Curzon–Ahlborn frontier, this yields $x=\tau^{3/4}$ and $\eta =1-\tau^{3/4}$. The plot is shown for τ=0.3.

The Newtonian CA frontier realizes the regular balanced case described in Section 8.2. Indeed, $R\left(x;0\right)=1-p\left(x\right)$ vanishes at the maximum-power point and increases toward the reversible edge, while $R\left(x;1\right)=q\left(x\right)$ vanishes at the reversible edge and increases toward the maximum-power point. The upper envelope of $\max\left\{1-p\left(x\right),q\left(x\right)\right\}$ is therefore minimized at the crossing of the two endpoint regrets (Figure 6). Hence,

(96) $$1-p\left(x_{reg}\right)=q\left(x_{reg}\right).$$

In terms of the fraction of exergy destruction avoided, i.e., $r\left(x\right)=1-q\left(x\right)$, the full-uncertainty minimax-regret condition is simply

(97) $$p\left(x_{reg}\right)=r\left(x_{reg}\right).$$

We now apply Equation (96) to the Newtonian CA frontier. Let $s=\sqrt{\tau }$. Using Equations (15), (16) and (80), the equal-regret condition becomes

(98) $$1-\frac{\left(1-x\right)\left(x-s^{2}\right)}{x{\left(1-s\right)}^{2}}=\frac{{\left(x-s^{2}\right)}^{2}}{sx{\left(1-s\right)}^{2}}.$$

Multiplying by $sx{\left(1-s\right)}^{2}$ and simplifying gives $x^{2}=s^{3}$. Since x>0, the physical solution is $x_{reg}=s^{3/2}=\tau^{3/4}$. Therefore, the regret-robust efficiency is

(99) $$\eta_{reg}=1-\tau^{3/4}.$$

This is precisely the Newtonian 3/4 law also selected by the AHAB $E_{\epsilon }$ − $C\left(\epsilon \right)$ prescription and by the symmetric bargaining selectors considered above. The present derivation gives it a distinct interpretation. The selected point is not obtained by prescribing a particular ecological weight. It is obtained by minimizing the worst-case regret under complete normalized uncertainty between two limiting priorities: retaining maximum power and minimizing exergy destruction.

In this form, the meaning of the 3/4 law is especially simple: the lost maximum-power fraction equals the remaining exergy-destruction burden. The operating point sacrifices exactly as much normalized maximum power as the normalized exergy-destruction burden it still retains. This equal-regret balance is the minimax-regret interpretation of this ecological operating state.

### 9.2. General Uncertainty Intervals

The full uncertainty interval $\theta \in \left[0,\;1\right]$ gives the most symmetric result, but the minimax-regret construction applies equally to any admissible uncertainty interval $\theta \in \left[\theta_{-},\theta_{+}\right]$, $0\leq \theta_{-}<\theta_{+}\leq 1$. Within the regular balanced case described in Section 8.2, the regret-robust operating point satisfies the equal-regret condition (90), where $K\left(\theta_{-},\theta_{+}\right)$ is defined by Equation (91). Equivalently, by the envelope relation, K is the average value of p+q along the fixed-θ optimal branch over the uncertainty interval.

For the Newtonian CA frontier, substituting the explicit expressions for p and q into Equation (90) gives, with $s=\sqrt{\tau }$,

(100) $$p\left(x\right)+q\left(x\right)=\frac{\left(x-s^{2}\right)\left(x+s\right)}{sx\left(1-s\right)}.$$

Therefore, $x_{reg}$ satisfies

(101) $$x_{reg}^{2}+s\left(1-s\right)\left(1-K\right)x_{reg}-s^{3}=0.$$

The physical root on the ecological branch is

(102) $$x_{reg}\left(K\right)=\frac{-s\left(1-s\right)\left(1-K\right)+\sqrt{s^{2}{\left(1-s\right)}^{2}{\left(1-K\right)}^{2}+4s^{3}}}{2}.$$

Thus, $\eta_{reg}\left(K\right)=1-x_{reg}\left(K\right)$. Since 0≤K≤2, this expression remains on the physical CA branch, between the reversible edge and the maximum-power point. The limiting cases are consistent: K=0 gives $x_{reg}=\tau$, K=1 gives $x_{reg}=\tau^{3/4}$, and K=2 gives $x_{reg}=\sqrt{\tau }$. If the uncertainty interval collapses to a single value, i.e., $\theta_{+}\to \theta_{-}=\theta_{0}$, the regret problem reduces to ordinary fixed-$\theta_{0}$ optimization, and $x_{reg}\to x^{⋆}\left(\theta_{0}\right)$. If the interval is the full range $\left[0,\;1\right]$, then K=1, and Equation (101) gives $x_{reg}=\tau^{3/4}$.

A useful special case is an uncertainty interval symmetric about equal normalized weighting, i.e.,

(103) $$\theta_{\pm }=\frac{1}{2}\pm \Delta ,\;\;\;\;\;\;0\leq \Delta \leq \frac{1}{2}.$$

For the CA frontier, this entire family gives the same regret-robust point as the full-uncertainty case. Indeed, the fixed-θ optimum satisfies

(104) $$x^{⋆}\left(1-\theta \right)=\frac{s^{3}}{x^{⋆}\left(\theta \right)},\;\;\;\;\;\;s=\sqrt{\tau },$$

while Equation (100) obeys the corresponding symmetry:

(105) $$\left[p+q\right]\left(\frac{s^{3}}{x}\right)=2-\left[p+q\right]\left(x\right).$$

Hence, the average of p+q over any interval symmetric about θ=1/2 is exactly unity, i.e., $K\left(\frac{1}{2}-\Delta ,\frac{1}{2}+\Delta \right)=1.$ Therefore, $x_{reg}=\tau^{3/4}$ and $\eta_{reg}=1-\tau^{3/4}$ for every symmetric uncertainty band around equal normalized priority. The 3/4 law is therefore not only the full-uncertainty solution but also the regret-robust solution for any symmetric uncertainty interval centered at θ=1/2.

For a proper partial interval, the regret-robust point lies between the two endpoint optima, i.e., $x^{⋆}\left(\theta_{+}\right)<x_{reg}<x^{⋆}\left(\theta_{-}\right)$, or, equivalently,

(106) $$\eta^{⋆}\left(\theta_{-}\right)<\eta_{reg}<\eta^{⋆}\left(\theta_{+}\right).$$

To illustrate this broader family, consider three uncertainty intervals of equal width: $\theta \in \left[0.1,\;0.5\right]$, $\left[0.3,\;0.7\right]$, and $\left[0.5,\;0.9\right]$. The first is shifted toward power retention, the second is symmetric about equal priority, and the third is shifted toward reduction in exergy destruction. For τ=0.5, the corresponding regret-robust efficiencies are $\eta_{reg}≃0.3630$, 0.4054, and 0.4450, respectively. The same trend persists as the reservoir temperature ratio is varied: for τ=0.25, the three values are approximately 0.5952, 0.6464, and 0.6912, whereas for τ=0.75, they are approximately 0.1706, 0.1941, and 0.2169. Thus, shifting the admissible priority interval toward a smaller θ moves the regret-robust state toward maximum-power operation, while shifting it toward a larger θ moves the state toward the reversible edge. The symmetric interval retains $\eta_{reg}=1-\tau^{3/4}$, whereas asymmetric intervals produce systematic departures on either side of this value. More generally, minimax regret does not select the optimum associated with a representative or midpoint value of θ; it selects the state that balances the losses relative to the best choices at the two extremes of the admissible interval. The full-uncertainty result $x_{reg}=\tau^{3/4}$ is therefore the symmetric member of a broader family of regret-robust operating states.

## 10. Discussion and Conclusions

The analysis above gives a unified decision-theoretic interpretation of ecological optimization in the endoreversible Curzon–Ahlborn engine. The physical model determines a feasible tradeoff between power output and exergy destruction, but it does not, by itself, select one operating state from the resulting Pareto frontier. A selection principle is still required. The main purpose of this work has been to distinguish clearly between the thermodynamic tradeoff itself, the normalized coordinates used to represent it, and the decision rule used to choose one point on it. Accordingly, the contribution is not the discovery of a new 3/4 efficiency law but a decision-theoretic interpretation of why this established intermediate operating point constitutes a distinguished compromise between maximum-power and reversible operation.

For Newtonian heat transfer, the symmetric Nash criterion selects $\eta_{N}=1-\tau^{3/4}$. This result is not merely an algebraic consequence of maximizing a particular product objective. In the normalized power–loss plane, it is the balanced point of the CA Pareto frontier: the retained fraction of maximum power equals the fraction of maximum-power exergy destruction that has been avoided. The 3/4 law therefore has a direct geometric and thermodynamic meaning.

This interpretation also explains why several symmetric selection principles coincide in the Newtonian CA model. The Nash product, the Kalai–Smorodinsky rule, and the egalitarian max–min rule all reduce to the same balance condition when the utilities are taken as normalized power retention and normalized reduction in exergy destruction. Thus, the Newtonian 3/4 law is not tied uniquely to the Nash product but reflects the special symmetric geometry of the normalized CA power–loss frontier. The selection principles nevertheless contain different information: Nash bargaining is characterized by the marginal product-of-gains condition (35), whereas Kalai–Smorodinsky and egalitarian selection reach the same Newtonian point through their respective proportional-gain and max–min criteria. Their coincidence at p=r therefore does not imply equivalence of the underlying rules.

The relation to the conventional weighted ecological family is equally transparent. The same Newtonian operating state can be represented as the optimum of $P-\lambda {\dot{X}}_{d}$, with the effective coefficient $\lambda^{⋆}=\tau^{-1/2}.$ The difference is interpretive. In the conventional weighted formulation, the coefficient must normally be prescribed from outside the thermodynamic optimization. In the Nash formulation, it emerges as the weighted-objective representation of a symmetric Pareto compromise.

This also clarifies the relation to the Arias-Hernández–Angulo-Brown prescription. Their $E_{\epsilon }$ family, together with the $C\left(\epsilon \right)$-selection procedure, reaches the same Newtonian operating point. In the language developed here, the two approaches therefore coincide in the Newtonian CA model, but this agreement does not imply equivalence of the underlying selection procedures. When the heat-transfer law is perturbed away from Newton’s law, the AHAB and Nash prescriptions follow different continuations and separate at first order in the transfer exponent. The resulting efficiency splitting is small near the Newtonian limit but systematic.

The bargaining formulation also gives a broader language for ecological optimization. Weighted Nash selection introduces a controlled priority asymmetry between power retention and irreversibility reduction. Externally specified nonzero disagreement points can represent a minimum acceptable power fraction, a required ecological improvement, or a status-quo operating mode.

The minimax-regret analysis addresses the same physical selection problem under a different informational condition. Bargaining optimization assumes that the relevant compromise structure is known before the operating state is chosen. The two objectives may be symmetric, their asymmetry may be specified, or a disagreement baseline may be given. Minimax regret applies when the relative priority between power retention and reduction in exergy destruction is itself uncertain. For complete normalized uncertainty, the result is particularly transparent. The regret associated with not operating at maximum power is the lost fraction of maximum power, whereas the regret associated with not eliminating exergy destruction is the remaining normalized exergy-destruction burden. Equalizing these two regrets again gives $\eta_{reg}=1-\tau^{3/4}$.

The Newtonian 3/4 law therefore acquires a second interpretation. It is not only a symmetric bargaining compromise but also a regret-robust operating point: it minimizes the worst loss caused by uncertainty about the relative importance of power and irreversibility reduction.

For smaller uncertainty intervals, minimax regret generates a broader family of regret-robust operating states. The full-uncertainty solution is the symmetric member of that family, and the same 3/4 law remains selected for every uncertainty interval symmetric about equal normalized priority. More generally, minimax regret does not simply choose the optimum associated with an average priority. It selects the operating state that balances the losses relative to the best choices at the two extremes of the admissible interval.

The main conceptual outcome is therefore a separation of roles. The physical engine model determines the feasible power–loss frontier. The thermodynamic coordinates determine what is being compared. The decision setting determines which selection principle is appropriate. Bargaining criteria select when the compromise structure is specified; minimax regret selects when that structure is uncertain. These are complementary rather than competing approaches.

The specific closed-form results obtained here pertain to the endoreversible CA model. In the Newtonian case, unequal hot- and cold-side conductances are already admitted and affect the dimensional scale but not the normalized power–loss frontier. More general heat-transfer laws [38], low-dissipation or non-endoreversible engine models [39,40,41], extensions of the endoreversible Carnot framework [7], and related systems with asymmetric thermal contacts [42] can modify the underlying performance frontier and therefore need not preserve the particular 3/4 operating point or the coincidence of the different symmetric selectors found here. The decision-theoretic procedure itself is more general: once the appropriate power–irreversibility Pareto frontier is constructed for a given physical model, bargaining or regret-based criteria can be applied to select an operating state from that frontier. Quantitative investigation of these broader models is left for future work.

## Data Availability

The data generated during this study are provided in the article.

## Appendix A. Derivation of Newtonian CA Power–Loss Pareto Branch

This appendix derives the reduced expressions used in the main text for the Newtonian endoreversible Curzon–Ahlborn engine. The purpose is to make explicit how the power curve, exergy-destruction curve, and normalized Pareto coordinates arise.

Consider an endoreversible engine operating between hot and cold reservoirs at temperatures of $T_{h}>T_{c}$. The working substance absorbs heat at internal temperature $T_{1}$ and rejects heat at internal temperature $T_{2}$, with $T_{h}>T_{1}>T_{2}>T_{c}.$

For Newtonian heat transfer,

(A1) $${\dot{Q}}_{h}=K_{h}\left(T_{h}-T_{1}\right),\;\;\;\;\;\;{\dot{Q}}_{c}=K_{c}\left(T_{2}-T_{c}\right).$$

Endoreversibility means that the internal cycle is reversible, so the entropies absorbed and rejected by the working substance are equal:

(A2) $$\frac{{\dot{Q}}_{h}}{T_{1}}=\frac{{\dot{Q}}_{c}}{T_{2}}.$$

We define $\tau =T_{c}/T_{h}$, $x=T_{2}/T_{1}$. The efficiency is then

(A3) $$\eta =1-\frac{{\dot{Q}}_{c}}{{\dot{Q}}_{h}}=1-\frac{T_{2}}{T_{1}}=1-x.$$

### Appendix A.1. Solving for the Heat Current

From Equation (A2),

(A4) $${\dot{Q}}_{c}=x\;{\dot{Q}}_{h}.$$

Using Equation (A1),

(A5) $$K_{c}\left(T_{2}-T_{c}\right)=xK_{h}\left(T_{h}-T_{1}\right).$$

Since $T_{2}=xT_{1}$, this becomes

(A6) $$K_{c}\left(xT_{1}-T_{c}\right)=xK_{h}\left(T_{h}-T_{1}\right).$$

Solving for $T_{1}$,

(A7) $$T_{1}=\frac{xK_{h}T_{h}+K_{c}T_{c}}{x\left(K_{h}+K_{c}\right)}.$$

The hot-side temperature difference is

(A8) $$T_{h}-T_{1}=T_{h}-\frac{xK_{h}T_{h}+K_{c}T_{c}}{x\left(K_{h}+K_{c}\right)}.$$

Using $T_{c}=\tau T_{h}$,

(A9) $$T_{h}-T_{1}=T_{h}\frac{K_{c}\left(x-\tau \right)}{x\left(K_{h}+K_{c}\right)}.$$

Thus,

(A10) $${\dot{Q}}_{h}=K_{h}\left(T_{h}-T_{1}\right)=T_{h}\frac{K_{h}K_{c}}{K_{h}+K_{c}}\frac{x-\tau }{x}.$$

We define

(A11) $$A=T_{h}\frac{K_{h}K_{c}}{K_{h}+K_{c}}.$$

Then,

(A12) $${\dot{Q}}_{h}=A\frac{x-\tau }{x}.$$

### Appendix A.2. Power Curve

The power output is

(A13) $$P={\dot{Q}}_{h}-{\dot{Q}}_{c}.$$

Using ${\dot{Q}}_{c}=x{\dot{Q}}_{h}$,

(A14) $$P=\left(1-x\right){\dot{Q}}_{h}.$$

Substituting Equation (A12),

(A15) $$P\left(x\right)=A\frac{\left(1-x\right)\left(x-\tau \right)}{x}.$$

The maximum-power point follows from dP/dx=0:

(A16) $$x_{MP}=\sqrt{\tau }.$$

The corresponding efficiency is

(A17) $$\eta_{MP}=1-\sqrt{\tau }.$$

At this point,

(A18) $$P_{MP}=A\frac{\left(1-\sqrt{\tau }\right)\left(\sqrt{\tau }-\tau \right)}{\sqrt{\tau }}=A{\left(1-\sqrt{\tau }\right)}^{2}.$$

### Appendix A.3. Exergy-Destruction Rate

The entropygeneration rate in the external reservoirs is

(A19) $${\dot{S}}_{gen}=-\frac{{\dot{Q}}_{h}}{T_{h}}+\frac{{\dot{Q}}_{c}}{T_{c}}.$$

Using ${\dot{Q}}_{c}=x{\dot{Q}}_{h}$ and $T_{c}=\tau T_{h}$,

(A20) $${\dot{S}}_{gen}=\frac{{\dot{Q}}_{h}}{T_{h}}\left(\frac{x}{\tau }-1\right)=\frac{{\dot{Q}}_{h}}{T_{h}}\frac{x-\tau }{\tau }.$$

Using $T_{0}=T_{c}$, the exergy-destruction rate is ${\dot{X}}_{d}=T_{c}{\dot{S}}_{gen}$. Substituting $T_{c}=\tau T_{h}$ into Equation (A20),

(A21) $${\dot{X}}_{d}={\dot{Q}}_{h}\left(x-\tau \right).$$

Using Equation (A12),

(A22) $${\dot{X}}_{d}\left(x\right)=A\frac{{\left(x-\tau \right)}^{2}}{x}.$$

At maximum power, $x=\sqrt{\tau }$, so

(A23) $${\dot{X}}_{d,MP}=A\sqrt{\tau }{\left(1-\sqrt{\tau }\right)}^{2}.$$

### Appendix A.4. Normalized Pareto Coordinates

The normalized retained-power coordinate is

(A24) $$p\left(x\right)=\frac{P\left(x\right)}{P_{MP}}.$$

Using Equations (A15) and (A18),

(A25) $$p\left(x\right)=\frac{\left(1-x\right)\left(x-\tau \right)}{x{\left(1-\sqrt{\tau }\right)}^{2}}.$$

The normalized reduction in exergy destruction is

(A26) $$r\left(x\right)=1-\frac{{\dot{X}}_{d}\left(x\right)}{{\dot{X}}_{d,MP}}.$$

Using Equations (A22) and (A23),

(A27) $$r\left(x\right)=1-\frac{{\left(x-\tau \right)}^{2}}{\sqrt{\tau }\;x{\left(1-\sqrt{\tau }\right)}^{2}}.$$

At the maximum-power point, $x=\sqrt{\tau }$, p=1, and r=0. At the reversible edge, x=τ, p=0, and r=1. Thus, the branch $x\in \left[\tau ,\sqrt{\tau }\right]$ is naturally represented as a Pareto curve connecting $\left(p,r\right)=\left(0,\;1\right)$ to $\left(p,r\right)=\left(1,\;0\right)$. This is the normalized power–loss frontier used throughout the main text.

## Appendix B. Perturbative Comparison of Nash and AHAB Selection for n=1+δ

This appendix compares two continuations away from Newtonian heat transfer:

1. The Arias-Hernández–Angulo-Brown (AHAB) ecological construction, based on the $E_{\epsilon }=P-\epsilon T_{c}{\dot{S}}_{gen}$ family, with ϵ selected by their $C\left(\epsilon \right)$ criterion;
2. The Nash ecological selector, which maximizes the product of normalized retained power and normalized reduction in exergy destruction.

The comparison is made in the same endoreversible CA setting with a generalized heat-transfer law:

(A28) $${\dot{Q}}_{h}=a{\left(T_{h}-T_{1}\right)}^{n},\;\;{\dot{Q}}_{c}=a{\left(T_{2}-T_{c}\right)}^{n},$$

where n=1+δ, $\left|\delta \right|\ll 1$.

### Appendix B.1. Reduced Model and Notation

Let $\tau =T_{c}/T_{h}$, x=1−η, and $t=\tau^{1/4}$. For the symmetric power-law heat-transfer model, we use the following reduced expressions:

(A29) $${\hat{P}}_{n}\left(x\right)=\frac{\left(1-x\right){\left(x-\tau \right)}^{n}}{x\;{\left[1+x^{\left(n-1\right)/n}\right]}^{n}}$$

and

(A30) $${\hat{\Sigma }}_{n}\left(x\right)=\frac{{\left(x-\tau \right)}^{n+1}}{x\;{\left[1+x^{\left(n-1\right)/n}\right]}^{n}}.$$

Here, ${\hat{P}}_{n}$ is proportional to the power output, while ${\hat{\Sigma }}_{n}$ is proportional to $T_{c}{\dot{S}}_{gen}$, i.e., to the exergy-destruction rate ${\dot{X}}_{d}=T_{c}{\dot{S}}_{gen}$. Overall positive prefactors are omitted because they do not affect maximizers.

The ratio

(A31) $$\frac{{\hat{P}}_{n}\left(x\right)}{{\hat{\Sigma }}_{n}\left(x\right)}=\frac{1-x}{x-\tau }$$

is independent of the heat-transfer exponent. This law-independent ratio is the reduced version of the endoreversible identity emphasized by AHAB.

### Appendix B.2. Maximum-Power Point

The maximum-power point is determined by $\partial {\hat{P}}_{n}\left(x\right)/\partial x=0$. Equivalently, we define

(A32) $$\Phi \left(x;n\right)=\ln{\hat{P}}_{n}\left(x\right).$$

Using Equation (A29),

(A33) $$\Phi \left(x;n\right)=\ln\left(1-x\right)-\ln x+n\ln\left(x-\tau \right)-n\ln\left(1+x^{\left(n-1\right)/n}\right).$$

The stationarity condition is

(A34) $$F\left(x;n\right)\equiv \partial_{x}\Phi \left(x;n\right)=0.$$

Explicitly,

(A35) $$F\left(x;n\right)=-\frac{1}{1-x}-\frac{1}{x}+\frac{n}{x-\tau }-\frac{\left(n-1\right)x^{-1/n}}{1+x^{\left(n-1\right)/n}}.$$

At n=1, one obtains the usual Newtonian CA maximum-power point $x_{MP}^{\left(0\right)}=\sqrt{\tau }=t^{2}$. Now, we expand

(A36) $$x_{MP}=t^{2}+\delta x_{MP}^{\left(1\right)}+O\left(\delta^{2}\right).$$

Implicit differentiation of $F\left(x;n\right)=0$ gives

(A37) $$x_{MP}^{\left(1\right)}=-{\left.\frac{F_{n}}{F_{x}}\;\right|}_{\;\left(x,n\right)=\left(t^{2},1\right)}.$$

A direct evaluation yields

(A38) $$x_{MP}^{\left(1\right)}=\frac{1-t^{4}}{4}.$$

Therefore,

(A39) $$x_{MP}=t^{2}+\delta \frac{1-t^{4}}{4}+O\left(\delta^{2}\right);$$

hence,

(A40) $$\eta_{MP}=1-t^{2}-\delta \frac{1-t^{4}}{4}+O\left(\delta^{2}\right).$$

For δ>0, the maximum-power efficiency is shifted downward relative to the Newtonian value.

### Appendix B.3. The AHAB $E_{\epsilon }$ − $C\left(\epsilon \right)$ Prescription and the Selected Weight

The Arias-Hernández–Angulo-Brown construction can be viewed as a two-stage selection procedure. The first stage introduces the following one-parameter family of ecological objectives:

(A41) $$E_{\epsilon }\left(x\right)=\hat{P}\left(x\right)-\epsilon \hat{\Sigma }\left(x\right),$$

where ϵ weights the exergy-destruction penalty. For each fixed value of ϵ, the corresponding operating point is

(A42) $$x_{\epsilon }=\arg\underset{x}{\max}E_{\epsilon }\left(x\right).$$

The second stage selects the best member of this family by means of a compromise function $C\left(\epsilon \right)$:

(A43) $$C\left(\epsilon \right)=\frac{\hat{P}\left(x_{\epsilon }\right)}{\hat{P}\left(x_{MP}\right)}-\frac{\hat{\Sigma }\left(x_{\epsilon }\right)}{\hat{\Sigma }\left(x_{MP}\right)},$$

where $x_{MP}$ is the maximum-power point for the same heat-transfer law. The AHAB prescription is therefore

(A44) $$\epsilon^{⋆}=\arg\underset{\epsilon }{\max}C\left(\epsilon \right),\;\;\;\;\;\;x_{AHAB}=x_{\epsilon^{⋆}}.$$

The following lemma shows that the C-selected weight is determined by the maximum-power state alone.

**Lemma A1.** *Assume that* $x_{\epsilon }$ *is a regular interior maximizer of* $E_{\epsilon }\left(x\right)$ *and that* $C\left(\epsilon \right)$ *is defined by Equation (A43). Then, the regular interior stationary point of* $C\left(\epsilon \right)$ *occurs at*

(A45) $$\epsilon^{⋆}=\frac{\hat{P}\left(x_{MP}\right)}{\hat{\Sigma }\left(x_{MP}\right)}.$$

*Under the same regularity assumptions, this stationary point is a local maximum of* $C\left(\epsilon \right)$.

**Proof of Lemma A1.** Since $x_{\epsilon }$ maximizes $E_{\epsilon }$, it satisfies

(A46) $$\frac{d}{dx}{\left[\hat{P}\left(x\right)-\epsilon \hat{\Sigma }\left(x\right)\right]}_{x=x_{\epsilon }}=0.$$

Therefore,

(A47) $${\hat{P}}^{\prime }\left(x_{\epsilon }\right)=\epsilon {\hat{\Sigma }}^{\prime }\left(x_{\epsilon }\right).$$

Differentiate $C\left(\epsilon \right)$ with respect to ϵ. Since $x_{MP}$, $\hat{P}\left(x_{MP}\right)$, and $\hat{\Sigma }\left(x_{MP}\right)$ are fixed with respect to ϵ,

(A48) $$\frac{dC}{d\epsilon }=x_{\epsilon }^{\prime }\left[\frac{{\hat{P}}^{\prime }\left(x_{\epsilon }\right)}{\hat{P}\left(x_{MP}\right)}-\frac{{\hat{\Sigma }}^{\prime }\left(x_{\epsilon }\right)}{\hat{\Sigma }\left(x_{MP}\right)}\right].$$

Using Equation (A47),

(A49) $$\frac{dC}{d\epsilon }=x_{\epsilon }^{\prime }{\hat{\Sigma }}^{\prime }\left(x_{\epsilon }\right)\left[\frac{\epsilon }{\hat{P}\left(x_{MP}\right)}-\frac{1}{\hat{\Sigma }\left(x_{MP}\right)}\right].$$

For a regular interior solution with $x_{\epsilon }^{\prime }\neq 0$ and ${\hat{\Sigma }}^{\prime }\left(x_{\epsilon }\right)\neq 0$, the condition dC/dϵ=0 gives

(A50) $$\epsilon^{⋆}=\frac{\hat{P}\left(x_{MP}\right)}{\hat{\Sigma }\left(x_{MP}\right)}.$$

To check that this stationary point is a maximum, define

(A51) $$F\left(x,\epsilon \right)={\hat{P}}^{\prime }\left(x\right)-\epsilon {\hat{\Sigma }}^{\prime }\left(x\right).$$

Since $F\left(x_{\epsilon },\epsilon \right)=0$, implicit differentiation gives

(A52) $$x_{\epsilon }^{\prime }=\frac{{\hat{\Sigma }}^{\prime }\left(x_{\epsilon }\right)}{\partial_{xx}E_{\epsilon }\left(x_{\epsilon }\right)}.$$

At a local maximum of $E_{\epsilon }$,

(A53) $$\partial_{xx}E_{\epsilon }\left(x_{\epsilon }\right)<0.$$

Differentiating Equation (A49) once more and evaluating at $\epsilon =\epsilon^{⋆}$ gives

(A54) $${\left.\frac{d^{2}C}{d\epsilon^{2}}\;\right|}_{\epsilon^{⋆}}=\frac{{\left[{\hat{\Sigma }}^{\prime }\left(x_{\epsilon^{⋆}}\right)\right]}^{2}}{\hat{P}\left(x_{MP}\right)\;\partial_{xx}E_{\epsilon^{⋆}}\left(x_{\epsilon^{⋆}}\right)}.$$

Since $\hat{P}\left(x_{MP}\right)>0$, the numerator is positive, and $\partial_{xx}E_{\epsilon^{⋆}}<0$, we have

(A55) $${\left.\frac{d^{2}C}{d\epsilon^{2}}\;\right|}_{\epsilon^{⋆}}<0.$$

Thus, $\epsilon^{⋆}$ is a local maximum of C. □

This result is independent of the particular heat-transfer law.

### Appendix B.4. Specialization to the Power-Law CA Model near n=1

Using the law-independent ratio (A31),

(A56) $$\epsilon^{⋆}\left(n\right)=\frac{1-x_{MP}}{x_{MP}-\tau }.$$

Substituting Equation (A39) gives

(A57) $$\epsilon^{⋆}\left(n\right)=t^{-2}-\delta \frac{{\left(1+t^{2}\right)}^{2}}{4t^{4}}+O\left(\delta^{2}\right).$$

Since $t^{2}=\sqrt{\tau }$, this may also be written as

(A58) $$\epsilon^{⋆}\left(n\right)=\tau^{-1/2}-\delta \frac{{\left(1+\sqrt{\tau }\right)}^{2}}{4\tau }+O\left(\delta^{2}\right).$$

At n=1, this reduces to $\epsilon^{⋆}\left(1\right)=\tau^{-1/2}$. This is the same effective weight that appears in the Newtonian Nash representation.

The AHAB operating point is obtained by maximizing $E_{\epsilon^{⋆}}\left(x;n\right)$ with respect to x. At n=1, this gives $x_{AHAB}^{\left(0\right)}=t^{3}$ and $\eta_{AHAB}^{\left(0\right)}=1-t^{3}$. Now, we write

(A59) $$x_{AHAB}=t^{3}+\delta A_{1}\left(\tau \right)+O\left(\delta^{2}\right).$$

Expanding the stationarity condition, i.e., $\partial E_{\epsilon^{⋆}}\left(x;n\right)/\partial x=0$, to first order in δ gives

(A60) $$A_{1}\left(\tau \right)=\frac{t}{8}\left(1+2t-2t^{3}-t^{4}\right).$$

Therefore,

(A61) $$\eta_{AHAB}\left(n\right)=1-t^{3}-\delta A_{1}\left(\tau \right)+O\left(\delta^{2}\right).$$

Because $A_{1}\left(\tau \right)>0$ for 0<τ<1, the AHAB-selected efficiency decreases below the Newtonian 3/4 law when δ>0.

### Appendix B.5. Nash Ecological Branch

The strict Nash continuation is defined differently. For each n, we recompute the normalized utilities as

(A62) $$p\left(x;n\right)=\frac{{\hat{P}}_{n}\left(x\right)}{{\hat{P}}_{n}\left(x_{MP}\right)},\;\;r\left(x;n\right)=1-\frac{{\hat{\Sigma }}_{n}\left(x\right)}{{\hat{\Sigma }}_{n}\left(x_{MP}\right)}.$$

The Nash ecological objective is $N\left(x;n\right)=p\left(x;n\right)r\left(x;n\right)$. The Nash-selected point satisfies $\partial N\left(x;n\right)/\partial x=0$. Equivalently, because N>0 in the interior of the ecological branch,

(A63) $$\frac{\partial }{\partial x}\ln N\left(x;n\right)=0.$$

At n=1, the Nash optimum is $x_{N}^{\left(0\right)}=t^{3}$ and $\eta_{N}^{\left(0\right)}=1-t^{3}$. For n=1+δ, we write

(A64) $$x_{N}=t^{3}+\delta N_{1}\left(\tau \right)+O\left(\delta^{2}\right).$$

A first-order expansion of the strict Nash stationarity condition (A63) gives

(A65) $$N_{1}\left(\tau \right)=\frac{t\left(1-t^{2}\right)}{8}\;\frac{B\left(t\right)}{D\left(t\right)},$$

where

(A66) $$B\left(t\right)=t^{6}+4t^{5}+5t^{4}+8t^{3}+5t^{2}+4t+1+2t^{2}{\left(1+t\right)}^{2}\ln\frac{{\left(1+t\right)}^{2}}{t},$$

(A67) $$D\left(t\right)=t^{4}+2t^{3}+4t^{2}+2t+1.$$

Thus,

(A68) $$\eta_{N}\left(n\right)=1-t^{3}-\delta N_{1}\left(\tau \right)+O\left(\delta^{2}\right).$$

The logarithmic terms in $N_{1}\left(\tau \right)$ originate from expanding $x^{\left(n-1\right)/n}=1+\delta \ln x+O\left(\delta^{2}\right)$, which appears inside ${\hat{P}}_{n}$ and ${\hat{\Sigma }}_{n}$. Their presence is therefore a feature of the strict Nash continuation applied to the n-dependent Pareto branch, not an artifact of using lnN for stationarity.

### Appendix B.6. Difference Between Nash and AHAB Selections

Equations (A61) and (A68) give

(A69) $$\eta_{N}-\eta_{AHAB}=\delta \left[A_{1}\left(\tau \right)-N_{1}\left(\tau \right)\right]+O\left(\delta^{2}\right).$$

For the symmetric model considered here, the $A_{1}\left(\tau \right)-N_{1}\left(\tau \right)$ coefficient changes sign once, at $\tau_{0}\approx 0.02458$. For $\tau >\tau_{0}$, the Nash continuation predicts a slightly higher efficiency than the AHAB continuation for δ>0, whereas for extremely large temperature contrasts ($\tau <\tau_{0}$) the ordering is reversed.

Thus, the two prescriptions agree exactly at Newtonian heat transfer, i.e., $\eta_{N}\left(1\right)=\eta_{AHAB}\left(1\right)=1-\tau^{3/4}$, but separate linearly when n=1+δ. The numerical size of the splitting is small, as expected for a perturbation around n=1, but it is systematic.
